# Smooth Muscle Tumor of Uncertain Malignant Potential (STUMP): A Systematic Review of the Literature in the Last 20 Years

**DOI:** 10.3390/curroncol31090388

**Published:** 2024-09-05

**Authors:** Carmen Elena Bucuri, Razvan Ciortea, Andrei Mihai Malutan, Valentin Oprea, Mihai Toma, Maria Patricia Roman, Cristina Mihaela Ormindean, Ionel Nati, Viorela Suciu, Dan Mihu

**Affiliations:** 1 2nd Department of Obstetrics and Gynecology, “Iuliu Hatieganu” University of Medicine and Pharmacy, 400012 Cluj-Napoca, Romania; cbucuriel@yahoo.com (C.E.B.); malutan_andrei@yahoo.com (A.M.M.); opreacv31@gmail.com (V.O.); mpr1388@gmail.com (M.P.R.); cristina.mihaela.prodan@gmail.com (C.M.O.); nati.ionel@yahoo.com (I.N.); suciuviorela@yahoo.com (V.S.); dan.mihu@yahoo.com (D.M.); 2 Clinical Department of Surgery, “Constantin Papilian” Emergency Clinical Military Hospital, 22 G-ral Traian Mosoiu, 400132 Cluj-Napoca, Romania; dr.mtoma.eru@gmail.com

**Keywords:** mitotic index, recurrence, tumor markers, uterine smooth muscle tumor

## Abstract

Smooth Muscle Tumor of Uncertain Malignant Potential (STUMP) is a rare uterine tumor primarily affecting perimenopausal and postmenopausal women, typically aged between 45 and 55 years. Characterized by ambiguous histological features, STUMPs present diagnostic challenges as they cannot be definitively classified as benign or malignant based on morphology alone. This systematic review aims to elucidate the clinical, pathological, immunohistochemical, and treatment-related characteristics of STUMPs through an analysis of the literature from the past 20 years. The study follows PRISMA guidelines, utilizing comprehensive searches of PubMed and Scopus databases, yielding 32 studies that meet the inclusion criteria. From the analysis of these studies, it was revealed that the clinical presentations vary from common symptoms such as abnormal uterine bleeding and pelvic pain to incidental detection of uterine mass. Histologically, STUMPs demonstrate features overlapping with both leiomyomas and leiomyosarcomas, including mild nuclear atypia, low mitotic indices, and focal necrosis. Immunohistochemical markers such as p16 and p53 have been investigated for prognostic significance. Elevated p16 expression, often associated with aggressive behavior, was observed in a subset of STUMPs. Surgical management, typically involving hysterectomy or tumorectomy, is the primary treatment, though the extent of resection is variable. Adjuvant therapies are not routinely recommended, but long-term surveillance is advised, especially for high-risk patients. Recurrence rates for STUMPs are approximately 12%, with factors such as high mitotic counts and coagulative necrosis indicating higher risk. This review highlights the complexity of STUMP diagnosis and management, emphasizing the need for more precise diagnostic criteria and individualized treatment strategies. Understanding the morphological, immunohistochemical, and clinical behavior of STUMPs can improve patient outcomes and guide future research in this diagnostically challenging area.

## 1. Introduction

Smooth muscle tumors of uncertain malignant potential (STUMPs) are rare tumors that occur in myometrium of the uterus and tend to affect perimenopausal or postmenopausal women aged between 45 and 55 years at diagnosis. Their complex presentation and symptoms make them difficult to diagnose definitively because their classification as benign or malignant cannot rely only on morphology alone. STUMPS account for approximately 2–5% of all uterine smooth muscle tumors and present difficult management decisions due to their uncertain malignant behavior [1]. While many STUMPs appear benign with favorable long-term outcomes, some may exhibit aggressive malignant potential, metastasize, and can increase the tumor-related mortality [2].

Several factors contribute to the classification difficulties presented by STUMPs. Histologically, STUMPs often demonstrate overlapping features between clearly benign and malignant uterine smooth muscle tumors [3]. Features such as mild nuclear atypia, low mitotic indices, or focal necrosis can make differentiating STUMPs from leiomyomas (L) or leiomyosarcomas (LMS) challenging. Immunohistochemical and molecular markers have been investigated to improve characterization, but heterogeneity exists, and prediction of clinical course remains problematic [4]. Additionally, STUMPs are generally treated surgically despite the fact that the required extend of resection isn’t standardized. More extensive surgery may improve oncological outcomes but is not always warranted given that many STUMPs demonstrate benign behavior.

The objectives of this study are to comprehensively review the current understanding of STUMPs, including clinical, pathological, immunohistochemical, and treatment-related characteristics. A better grasp of defining features, prognostic indicators, and impact of surgical management strategies may help optimize patient classification and care. This paper will discuss morphological criteria used to diagnose STUMPs, investigate molecular markers with potential prognostic significance, and analyze treatment approaches and long-term outcomes reported in the literature. Identifying the means to accurately predict malignant potential in STUMPs could help guide management decisions for these diagnostically enigmatic uterine tumors.

## 2. Materials and Methods

**Figure 1:**
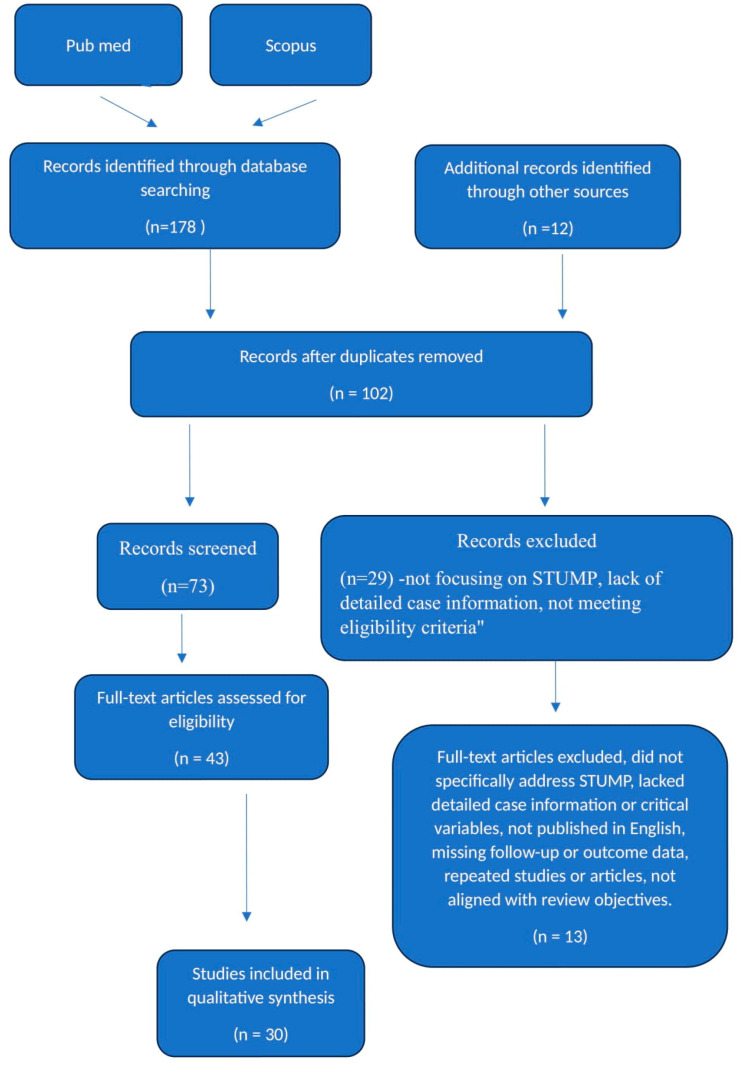
Systematic review flow diagram. Caption: the PRISMA flow diagram for the systematic review detailing the database searches, the number of abstracts screened, and the full texts retrieved.

This systematic review was conducted according to the Preferred Reporting Items for Systematic Reviews and Meta-Analyses (PRISMA) guidelines, Supplementary Materials. This protocol review was registered on the INPLASY platform, which stands for International Platform of Registered Systematic Review and Meta-Analysis Protocols. The protocol has been registered with the code INPLASY202460100. A comprehensive search of the PubMed and Scopus databases was performed to identify all the relevant literature published between January 2003 and December 2023. For PubMed, the search query used was “uterine smooth muscle tumor of uncertain malignant potential” in any field. For Scopus, the same phrase was entered into the title, abstract, and keyword fields. Only articles written in the English language were included based on the eligibility criteria. The initial search yielded a total of 178 records across the two databases. After removing duplicate articles, the remaining 102 titles and abstracts were screened to determine their relevance to the objectives of the review. Articles not focusing on STUMPs or lacking detailed case information were excluded at this stage. The full texts of the 43 potentially eligible articles were then assessed for inclusion. Reference lists of included studies were also manually searched to identify any other relevant publications. This process led to the final inclusion of 30 studies that satisfy the requirements of reporting complete case descriptions diagnosed as STUMP according to the Stanford pathological criteria. Data were then extracted from these 30 articles that thoroughly described the clinical, pathological, and treatment details of reported STUMP cases (Figure 1).

The selection criteria completeness: Only those cases that had sufficient follow-up data were included. This was crucial for assessing long-term outcomes and recurrence risks, which are central to the review. Reports lacking adequate follow-up data were excluded. Exclusion criteria: Studies were excluded if they focused on conditions other than STUMP or did not provide detailed information necessary for a thorough analysis. Studies that included broader categories of uterine smooth muscle tumors without specific focus on STUMP or lacked detailed clinical and pathological data were also excluded.

Each article was screened by a team of three reviewers to ensure accuracy and consistency. The initial screening of titles and abstracts was conducted by two reviewers independently, followed by a full-text review by the same team to confirm eligibility based on the inclusion criteria.

The risk of bias for each study was assessed using the Newcastle–Ottawa Scale (NOS), a tool designed to evaluate the quality of non-randomized studies in meta-analyses. The NOS assesses studies based on three broad categories: selection of the study groups, comparability of the groups, and ascertainment of either the exposure or outcome of interest. Each study was evaluated and assigned a score, which contributed to the overall assessment of study quality and the reliability of the findings. Studies with a high risk of bias were carefully considered in the interpretation of results, particularly regarding their influence on the overall conclusions of this review.

## 3. Results and Discussion

### 3.1. Diagnosis and Presentation

In the 30 reviewed articles (Table 1), a total of 153 cases were mentioned, however, of those only 99 cases contained sufficient information for an in-depth analysis. The mean age of the patients in the reported cases was 53 years and out of the 30 articles, 12 discussed recurrence. There was a total of 12 cases of recurrence across all the cases studied cumulatively, which is a rough percentage of 12%. In our systematic review, we focused specifically on 153 cases out of the 651 total patients mentioned across various studies because these cases were directly relevant to our review’s objectives. The selection criteria for inclusion were stringent, emphasizing studies and patient cases that provided comprehensive data on the key aspects we aimed to investigate, such as the histopathological features, clinical outcomes, and recurrence rates associated with STUMP.

The quality of the included studies was assessed using the Newcastle–Ottawa Scale. The studies showed varying levels of methodological rigor, with NOS scores ranging from 5 to 8 out of a possible 9. The median NOS score across the reviewed studies was 7, indicating a moderate to high quality of evidence overall. This variation in study quality underscores the need for cautious interpretation of the findings, particularly regarding their generalizability.

Many of the other cases, while informative, did not align precisely with our study parameters. For instance, some studies included patients with conditions other than STUMP, or they focused on broader categories of uterine smooth muscle tumors without the detailed focus necessary for our analysis. Additionally, some reports lacked sufficient follow-up data, making them less suitable for assessing long-term outcomes and recurrence risks, which are central to our review.

Common presentation for STUMPs includes symptoms such as abnormal uterine bleeding, pain in the pelvic area, and a sensation of pelvic pressure, or an incidental finding of a uterine mass on imaging or examination. Many STUMPs remain asymptomatic and are detected during evaluation for an unrelated condition. On gross examination, STUMPs vary in size from 1–20 cm and can appear quite similar to L or LMS [4]. The diagnosis of a STUMP tumor is usually made postoperatively following a hysterectomy or myomectomy performed for presumed fibroids. This often occurs when the pathological examination reveals atypical features not consistent with benign fibroids [5]. The identification of STUMP tumors relies on histological analysis, which may show characteristics that are intermediate between benign L and LMS [4].

The classical criteria introduced by Dall’Asta et al. (2014) also provide important guidelines. These criteria emphasize the cellular atypia being more pronounced than in typical L, the mitotic index being elevated but below the threshold for malignancy, the necrosis and hemorrhage being absent or minimally present [6].

We cannot overlook the important role of medical imaging tools, including ultrasound, MRI, and CT scans, which play a crucial, though limited, role in the diagnosis and management of STUMPs. These modalities are generally not able to distinguish STUMPs from benign L definitively. The diagnosis of STUMPs is typically made postoperatively based on histopathological examination of the surgical specimen. Ultrasonography may show features suggestive of L but cannot reliably differentiate STUMPs from benign L due to overlapping sonographic features. Similarly, MRI and CT scans may reveal features suggestive of L (such as well-defined margins and homogeneous enhancement) but do not provide specific features that distinguish STUMPs [6].

Immunohistochemical staining is another tool used in diagnosing STUMP: progesterone receptor (PR) is often positive in STUMPs, reflecting hormonal influence; Smooth Muscle Actin (SMA) and Caldesmon are typically positive, indicating smooth muscle differentiation; Desmin is positive in most cases, supporting smooth muscle lineage; Cytokeratin AE 1/3 (CK AE 1/3) is usually negative, which is used to rule out epithelial components; and CD 10 has a variable expression, being positive in some cases [6].

Management of STUMPs is demanding due to the uncertain malignant potential. This condition is commonly addressed through surgery even though the adequacy of surgery depends on factors like tumor size, depth of invasion of the myometrium, and cervical involvement [2,5]. Adjuvant treatments are not routinely recommended, and their usage will be determined on a case basis for STUMPs displaying higher risk features. Long-term surveillance consisting of clinical exams and imaging every 6–12 months for at least 5 years is advised for high-risk patients due to the risk of metastasis or recurrence.

In the study by Dall’Asta et al. (2014) on uterine STUMP pathology the researchers investigate five cases of uterine masses treated using surgical procedures to ascertain the factors that dictate patient outcomes and recurrence rate. According to this study, the diagnosis of STUMP is considerably more difficult in pre-menopausal women with fertility intensions [6]. Factors like cellularity and tumor borders, which are used in the diagnosis of STUMP, are “subjective” and can lead to interpretive differences among pathologists and STUMP can present with features that overlap with both L and LMS, making a definitive diagnosis laborious [1,5]. The ambiguous nature of STUMP has resulted in an “overdiagnosis of this neoplasia” over time, suggesting that STUMP may be misclassified in some cases [7]. L generally follow a benign clinical course, whereas LMS exhibit aggressive malignancy and often prove fatal. Conversely, STUMPs have an erratic clinical course, making their diagnosis and treatment challenging [8,9].

In another study carried out by Ip et al. (2009) involving a clinicopathological analysis of 16 cases of STUMP, the researcher begins with a simplistic definition of STUMP as any uterine smooth muscle tumor that cannot be definitively diagnosed as benign or malignant. However, it provides a more detailed view on the identification and potential classification of STUMPs into four fundamental subgroups. The first subgroup is “Atypical leiomyoma with limited experience” (AL-LE), the second subgroup is “Smooth muscle tumor of low malignant potential” (SMT-LMP), the third is “Atypical leiomyoma, low risk of recurrence” (AL-LRR), and the last group is “Mitotically active leiomyoma, limited experience” (MAL-LE) [10]. These subgroups are established using criteria based on the criterion of an assessment of atypia, mitotic rate, and presence/absence of tumor cell necrosis. In the sixteen cases featured in the article by Ip et al. (2009), six cases were AL-LE, seven cases were SMT-LMP, two cases were AL-LRR, and one case was MAL-LE [10]. Patient ages ranged from 25 to 64 years, with a median age of 48 years, and the most common presenting symptoms were abnormal uterine bleeding (AUB) and pelvic/abdominal mass. However, the authors of this article emphasize the limitations regarding the diagnosis, including inherent difficulties and subjectivity in the interpretation of atypia, mitotic rate, and tumor cell necrosis and the lack of uniform diagnostic criteria which can lead to diagnostic uncertainty and potential overdiagnosis of STUMPs and the lack of uniform diagnostic criteria [10,11].

### 3.2. Comparison with Leiomyomas and Its Variants

O’Neill et al. (2007) indicated that there was no significant difference in p16 expression between usual L, leiomyoma variants, and STUMPs [12,13,14]. This suggests that these three groups of uterine smooth muscle tumors share some similarities in terms of the underlying molecular mechanisms, as they do not exhibit overexpression of the p16 protein that is characteristic of leiomyosarcomas [15].

P16 and p53 are important genes to be taken into consideration in the assessment of STUMP cases and their recurrence [12]. P16 is a tumor suppressor protein that regulates cell cycles; however, its overexpression can paradoxically lead to tumor formation. When it is highly present, it can be associated with a more aggressive tumor biology. On the other hand, p53 is a well-known tumor suppressor gene that plays a crucial role in regulating cell growth and division [6,12]. According to Atkins et al. (2008), p16 expression can help distinguish between uterine smooth muscle tumors since they found that the majority (12 of 15) leiomyosarcomas (LMS) exhibited strong, diffuse p16 positivity, unlike leiomyomas where only three of twenty-two showed focal staining. For STUMPs, which can be very difficult to classify accurately, three of the eight cases developed metastatic disease. Significantly, the two STUMPs that metastasized displayed strong, diffuse p16 expression. While these two tumors only showed mild cytologic atypia, they were characterized by the presence of coagulative tumor cell necrosis [1]. This suggests that in STUMPs exhibiting necrosis as well as high p16 levels, the tumor should potentially be reclassified as an LMS rather than a STUMP, given the association with aggressive metastatic behavior. These findings reinforce that p16 is preferentially expressed in LMS versus L, and that p16 immunohistochemistry could aid in the pathological assessment of diagnostically challenging uterine smooth muscle tumors, helping to better distinguish them and perhaps identify those STUMPs more likely to behave in a malignant fashion. This is supported by the study by Bodner-Adler et al. (2005) indicating that p16 could improve classification of these tumor categories [12].

Leiomyoma variants, such as symplastic (atypical), mitotically active, and cellular/highly cellular L, can sometimes resemble LMS due to features like nuclear atypia, high mitotic index, and high cellularity [14]. Nonetheless, the results of Berretta (2008) showed that in terms of p16 expression, STUMPs did not significantly differ from usual L and leiomyoma variants. As p16 expression helped distinguish clearly benign from malignant tumors, the similar p16 profile of STUMPs compared to the benign tumors suggests that STUMPs may also be part of the benign end of the spectrum, despite their designation as uncertain [16]. This highlights the challenge in accurately predicting the aggressive nature of some of these borderline STUMPs based solely on morphological evaluation, as some may not follow expected benign behavior like typical L and variants [15]. While STUMPs are defined as uncertain based on morphology, ancillary markers like p16 may provide some clues, though following clinical behavior over time is still important to fully characterize these problematic tumors [17].

In O’Neill et al. (2007), P16 expression was found to be significantly different between LMS and other tumor groups. Nineteen of twenty-two (86%) LMS exhibited strong diffuse p16 positivity (>50% of cells staining), compared to very few other tumors. There was a statistically significant difference in both p16 staining distribution (*p* < 0.001) and intensity (*p* = 0.001) between LMS and the combined group of usual LMS, variants, and STUMPs [14]. While some leiomyoma variants and one STUMP showed strong p16 expression, diffuse high levels were only seen in LMS a defining distinction that suggests p16 could help diagnose problematic cases [14,18].

Moreover, the expression of p53 also notably differed, with seven of twenty-two (32%) LMS showing strong nuclear positivity in over 50% of tumor cells, a statistically significant difference compared to other groups (*p* = 0.014). Only one leiomyoma variant exceeded 50% positivity. Conversely, all usual L and STUMPs had low p53 labeling indices of 0–2+, indicating p53 positivity is specific but lacks sensitivity for malignancy on its own. MIB1 proliferation rates showed the most definitive separation, with nineteen of twenty-two (86%) LMS exceeding an index of 20%, and a mean proliferation rate of 49.5% compared to under 7–7.5% for other groups (*p* < 0.001) [14]. This strongly supports the highly proliferative nature of LMS versus other tumors. While not perfectly sensitive alone, the combination of elevated p16, p53, and MIB1 expression appears to confirm malignancy in problematic uterine smooth muscle tumors [19].

### 3.3. Tumor Characteristics

Regarding the tumor size and characteristics, Ip at al (2009) noted that tumors associated with uterine STUMP ranged between 0.7 cm and 18 cm [10]. According to Dall’Asta et al. (2014), tumors typically measure approximately 10 cm to 12 cm in diameter, as evidenced by case studies of the featured patients [6]. The investigation of the mitotic activity revealed that the mitotic counts in the STUMP cases ranged from four to five mitotic figures per ten high-power fields. A majority of the tumors were larger than 5 cm and most tumors were located in the uterine corpus (intramural, IM), with a few in the uterine wall (subserosal, SS) or cervix (SM). Cellularity was generally described as diffuse and moderate to high (grade 2–3) and cytological atypia ranged from focal to multifocal and mild to severe [10]. In the immunohistochemistry analysis, p16 and p53 staining was variable, with diffuse strong positivity in the two recurrent AL-LE cases and MIB-1 (Ki-67) labeling index ranged from less than 5% to 25%. MIB-1 (Ki-67) is a widely used immunohistochemical marker that measures cellular proliferation [20]. It is a nuclear antigen expressed in proliferating cells during all active phases of the cell cycle (G1, S, G2, and M) but is absent in resting (G0) cells. Estrogen and progesterone receptor expression was generally positive, with variable intensity [10].

The mitotic index, defined as the number of mitotic figures per ten high-power fields, is another key criterion used to assess the biological potential of uterine smooth muscle tumors [21]. Also, coagulative tumor cell necrosis is the single most powerful factor for malignant behavior, which is characterized by an abrupt transition between viable cells and an area of necrosis and should be differentiated from other types of innocuous necrosis [13,22]. Significant (moderate to severe) atypia characterized by the presence of pleomorphism and nuclear hyperchromasia is an important feature. Nonetheless, the growth patterns of STUMP tumors are generally unpredictable and vary widely among patients [23].

STUMP exhibits distinctive molecular characteristics crucial for understanding its behavior and guiding treatment. p16 is a tumor suppressor protein often overexpressed in STUMP tumors, indicating abnormal cell cycle regulation. p53 is a critical tumor suppressor that can show variable expression, from weak to strong, in STUMP cases. Elevated p53 levels often suggest mutations impacting its function, contributing to tumor development. Ki-67 (MIB-1) indicates the percentage of actively dividing cells. In STUMP tumors, the Ki-67 labeling index ranges from less than 5% to 25%, reflecting a spectrum of tumor proliferation rates. Higher Ki-67 levels are associated with more aggressive tumor behavior. Estrogen Receptors (ER) and Progesterone Receptors (PR) are generally positive in STUMP tumors. Variable expression intensity suggests that hormonal influences may play a role in tumor growth. This variability may impact treatment options, with hormonal therapies considered based on receptor status.

Overall, the molecular profile of STUMP tumors, including p16 and p53 expression, Ki-67 labeling, and hormone receptor status, provides essential insights into tumor biology and potential therapeutic strategies. The variability in these markers necessitates personalized management approaches to effectively address STUMP cases [24,25,26].

### 3.4. Treatment

Based on the findings, it can be established that abdominal hysterectomy, with or without salpingo-oophorectomy is one of the most efficient approaches to the removal of STUMPs. In Dall’Asta et al. (2014), four patients underwent this surgical approach while one patient underwent excision of the uterine mass followed by total abdominal hysterectomy plus bilateral salpingo-oophorectomy after the STUMP diagnosis [6]. According to Shapiro et al. (2004) [25], most STUMPs are initially treated surgically with tumorectomy or hysterectomy, depending on factors like tumor location and size.

However, there are significant limitations and uncertainties regarding the optimal treatment. Shapiro et al. (2004) indicate that adjuvant treatment beyond surgery alone is not usually recommended for STUMPs, but may be considered on an individual basis depending on risk factor profiles [25].

While hysterectomy is often the preferred initial surgical treatment for STUMPs due to their uncertain malignant potential, myomectomy alone may be a reasonable option for selected young patients wishing to preserve fertility. Due to the generally favorable prognosis even for higher-risk STUMP subtypes, complete surgical excision via myomectomy could be adequate therapy for early-stage lesions in women who have not completed childbearing [27]. There is variability in clinical outcomes, and we emphasize the need for individualized treatment plans. There is a lack of definitive evidence for the benefit of prophylactic salpingo-oophorectomy in STUMP cases and there is no consensus if this procedure should be performed [5]. Close follow-up would still be recommended given the risk of late recurrence. However, for those seeking future pregnancies, myomectomy allows for preservation of reproductive function. Younger age and desire for fertility would weigh in favor of attempting uterus-sparing surgery in appropriately counseled patients [27]. Of course, factors like tumor size, depth of invasion, and the possibility of complete excision are to be carefully taken into consideration in such cases. Overall, while data regarding long-term outcomes are still limited, myomectomy could be considered a valid alternative to hysterectomy for some young, low-risk STUMP patients hoping to maintain fertility options in the short term.

Another approach that has been recommended for clinical practice is morcellation [28]. This refers to the surgical technique used to remove large masses through small incisions, more specifically cutting a large tissue sample into smaller pieces so it can be withdrawn through the incision or a cannula. This approach comes with some of the benefits of minimally invasive surgery mainly because laparoscopic procedures for large uterine STUMPs that may otherwise require open abdominal surgery result in smaller incisions and reduce recovery time. It also helps preserve fertility by avoiding full hysterectomy in young patients and instead performs minimally invasive myomectomy [28]. However, there is a possibility that morcellating an undiagnosed STUMP or early-stage sarcoma could spread possibly malignant cells through the abdominal cavity, increasing risk of recurrence or metastases. Also, morcellation may make further surgical staging or treatment more difficult if malignancy is confirmed after.

There is no standardized prophylactic method specifically for the prevention of recurrence or metastasis of STUMP tumors, primarily due to the limited evidence and variability in clinical outcomes.

Limited but promising evidence suggests that hormonal therapy, such as progestins or GnRH agonists, could be beneficial for managing STUMP tumors. These treatments might help control tumor growth and recurrence in PR-positive cases [4].

### 3.5. Recurrence

The recurrence of uterine STUMP is relatively low, and it is influenced by a wide range of factors. Although these tumors do not carry a high malignant potential, 12 of the reviewed studies described recurrence and metastases. The histopathological features of the tumor can provide clues about its recurrence potential. STUMPs that express higher grade features such as marked nuclear atypia, higher mitotic activity, and tumor necrosis are more likely to recur than those with less severe abnormalities. The presence of infiltrative tumor borders is also associated with increased recurrence risk. Ip, Tse, and Tam (2010) found that STUMPs with coagulative tumor cell necrosis had a significantly higher recurrence rate compared to those without necrosis [29]. Immunohistochemical markers like elevated p16 and p53 expression levels may also indicate higher risk, as these tumors are more likely to exhibit malignant behavior [30].

For the patient whose STUMP recurred as a high-grade leiomyosarcoma in the humerus bone over 5 years after the initial treatment, the solitary nature and distant site of recurrence led physicians to opt for wide excision of the proximal humerus only, reconstructed with endoprosthesis, as chemotherapy was deemed of limited efficacy [25]. However, additional lung metastases were discovered just one-year post-operation, highlighting the potential for recurrence and spread even with aggressive local intervention. This case therefore underscores the unpredictable behavior and the challenges in determining appropriate long-term management approaches of STUMPs.

The bivariate analysis of the total cases reviewed in the featured articles revealed the following findings. High mitotic count vs. low/normal mitotic count: there were three cases with a high mitotic count (>10/10HPF), among which one case experienced recurrence (33.3% recurrence rate). In contrast, there were 96 cases with low/normal mitotic counts, with seven cases experiencing recurrence (7.3% recurrence rate). The Fisher’s Exact Test yielded a *p*-value of 0.116, indicating that the difference in recurrence rates between high and low/normal mitotic counts was not statistically significant. Regarding the severe cytologic atypia vs. mild/moderate atypia: there were twelve cases with severe cytologic atypia, among which one case experienced recurrence (8.3% recurrence rate). For mild/moderate cytologic atypia, there were eighty-seven cases, with seven cases experiencing recurrence (8.0% recurrence rate). The Fisher’s Exact Test yielded a *p*-value of 1.000, indicating that the difference in recurrence rates between severe and mild/moderate cytologic atypia was not statistically significant. Regarding the presence vs. absence of coagulative necrosis: there were eight cases with coagulative necrosis present, among which one case experienced recurrence (12.5% recurrence rate). In cases without coagulative necrosis (91 cases), there were seven cases experiencing recurrence (7.7% recurrence rate). The Fisher’s Exact Test yielded a *p*-value of 0.691, indicating that the difference in recurrence rates between cases with and without coagulative necrosis was not statistically significant. In summary, the bivariate analysis did not find statistically significant associations between high mitotic count, severe cytologic atypia, or the presence of coagulative necrosis with recurrence rates in the reviewed cases. This suggests that these factors alone may not reliably predict recurrence risk in patients with STUMPs, based on the analyzed data.

In the study by Dall’Asta et al. (2014), all 5 patients were in the study remained recurrence-free during the follow-up period, which ranged from 6 to 81 months. It was however noted that the recurrence rates for STUMP are estimated to be between 8.7% and 11% and may include delayed recurrences. The diagnosis of uterine STUMP is subjective, so the best way to prevent short- and long-term recurrence is unclear. Experts recommend using immunohistochemistry to look at increased levels of p16 and p53, as this may help find patients more likely to have a recurrence who could benefit from more aggressive surgery and follow up [6]. Dall’Asta et al. (2014) found that preventing STUMP recurrence may involve reducing p16 and p53 [6]. Ip et al. (2009) supported this, finding that stains for p16, p53, MIB1 (Ki-67), and hormone receptors on two tumors that recurred showed strong p16 and p53, while six others without recurrence only had these in some areas [10].

Recurrence can also be contributed to by surgical factors. STUMPs cannot be definitively classified, which raises the question of which is the ideal surgical approach. This topic remains a subject of debate. It can be stated that more extensive surgery appears to lower recurrence rates. Studies have found lower recurrence for STUMPs treated with hysterectomy compared to tumorectomy. Complete surgical resection with negative margins is important. For large tumors, a simple tumorectomy may not remove the entire neoplasm, increasing recurrence risk. Radical surgery like hysterectomy reduces the amount of residual tissue harboring potential malignant foci. Lymphadenectomy is not routinely recommended but may be considered for higher risk STUMPs. Incomplete surgery and positive margins are associated with a greater risk of local recurrence after surgery for STUMP.

It is also important to consider the risk of metastasis, which can occur in some cases of tumors with high potential for malignancy. According to Shapiro et al. (2004), while most STUMPs behave in a benign manner, some can display a malignant clinical course with recurrence and metastasis. Features associated with metastasis in other studies include a mitotic index of over 10 mitoses per 10 HPFs, diffuse cytologic atypia, and coagulative tumor cell necrosis (CTCN) [25]. One study cited subdivided STUMPs and found that forty-six women with lesions characterized by diffuse, moderate to severe cytologic atypia and mitotic index under ten per ten HPFs experienced benign outcomes, except for one case of malignant behavior. Another STUMP type from the same study included five women with focal, severe cytologic atypia and mitotic index under twenty per ten HPFs, who all had benign outcomes. A third STUMP group referenced contained four cases, one of which (25%) experienced a malignant clinical course, emphasizing the importance of coagulative tumor cell necrosis [25].

### 3.6. Improving Patient Outcomes

Dall’Asta et al. (2014) emphasized the need for a multidisciplinary management involving gynecologists, dedicated gynecological pathologists, and oncologists to carefully counsel and manage patients diagnosed with STUMP, given the uncertainties around its malignant potential, diagnostic criteria, optimal treatment, and appropriate follow-up [6]. According to Ip et al., (2009), patient follow-up is very critical after diagnosis and treatment of STUMP. For the 16 cases that were featured in the study, the mean follow-up was 80.8 months and the median was 51.5 months. With the follow-up schedule, only two out of the sixteen tumors recurred, at 15 and 51 months after the initial surgery and patients with recurrence were alive at the last follow-up (40 and 74 months). The rest of the cases were successfully managed, and the patient remained alive and disease-free [10]. Shapiro et al. (2004) also advocate long-term follow up even for STUMPs lacking aggressive features given the unpredictability in clinical behavior. The average follow-up time until recurrence of the case presented was reported as 51 months (over 5 years) post-initial hysterectomy [25]. Toledo, G., and Oliva suggest that close postoperative monitoring is crucial given the unpredictable behavior of STUMP tumors. No standardized surveillance protocol exists, but regular follow-ups combining clinical and imaging assessments are advised. We emphasize individualized surveillance plans based on patient-specific risk factors and clinical findings, highlighting the importance of early detection through regular monitoring [4].

Based on our review of the literature, we propose the following recommendations for the management of uterine STUMP:

Multidisciplinary approach: Engage a team of gynecologists, dedicated gynecological pathologists, and oncologists to ensure comprehensive patient care. This approach is crucial due to the variability in STUMP’s malignant potential and the complexity of its diagnosis and treatment [6].

Individualized follow-up: Implement personalized follow-up plans that include regular clinical and imaging assessments. Given the unpredictable behavior of STUMP tumors, monitoring should be tailored to individual risk factors and clinical findings. Regular follow-up is essential to detect potential recurrences early and manage them effectively [4,10].

Long-term surveillance: Even in cases lacking aggressive features, long-term follow-up is recommended. Studies suggest that recurrences may occur many years after initial treatment, so continuous surveillance is critical to ensure timely intervention if needed [25].

Regarding the areas for further research, there is a need for developing standardized follow-up protocols to guide clinical practice. Research should focus on creating evidence-based guidelines for monitoring STUMP patients to improve consistency in care. Further investigation into molecular and histological markers that could better predict the behavior of STUMP tumors is warranted. Understanding these markers could aid in distinguishing between benign and malignant cases and tailor treatment strategies accordingly. More studies are needed to evaluate the efficacy of various treatment modalities and adjuvant therapies for STUMP tumors. This research could help identify optimal treatment strategies and improving patient outcomes.

Risk of bias:

The results of our risk of bias assessment indicated varying levels of bias across the included studies. We used the Newcastle–Ottawa Scale (NOS) to evaluate the risk of bias. The assessment revealed that the series of case reports were assessed as having a moderate to high risk of bias due to several factors: the lack of control groups, small sample sizes, and potential for selective outcome reporting. These limitations underscore the need for cautious interpretation of the findings, particularly regarding the generalizability of the conclusions drawn from these studies. The high risk of bias in some of the included studies suggests that the current evidence base for STUMP is limited by methodological constraints. This limitation affects the strength of the evidence and highlights the need for well-designed, larger studies that can provide more robust and generalizable data. Future research should aim to minimize bias through improved study design, including prospective data collection, standardized diagnostic criteria, and long-term follow-up. Incorporating these practices will enhance the reliability of the findings and contribute to a more comprehensive understanding of STUMP.

**Table 1:** Clinical features of patients included in the analyzed papers.

References	Number of Patients	Age Range (Years)	Recurrence (Yes/No)	Histology	Treatment	Follow-Up (Months)
Croce et al. (2015) [20]; Deodhar et al. (2011) [2]; Guntupalli et al. (2009); Ip et al. (2009) [10]; McCarthy and Chetty (2018) [3]; Toledo and Oliva (2008) [4]	157	24–68	Yes: 17, No: 140	Atypical cells, variable mitotic activity, nuclear atypia	Hysterectomy, myomectomy	6–60
Atkins et al. (2008) [8]; Bodner-Adler et al. (2005) [12]; Chen and Yang (2008) [7]; Hewedi et al. (2012) [11]; O’Neill et al. (2007) [14]; Shapiro et al. (2004) [25]	126	30–62	Yes: 16, No: 110	p16, p53, Ki-67 expression	Hysterectomy, myomectomy	6–60
Akbarzadeh-Jahromi et al. (2024) [26]; Dall’Asta et al. (2014) [6]; Ip and Cheung (2011) [19]; Ip et al. (2010) [29]; Mowers et al. (2015) [28]; Richtarova et al. (2023) [27]; Ünver et al. (2011) [18]	162	28–64	Yes: 20, No: 142	Cellular atypia, mitotic count, variable histology	Hysterectomy, myomectomy	6–60
Amant et al. (2005) [13]; Bacanakgil et al. (2017) [30]; Cohen et al. (2007) [21]; Gadducci and Zannoni (2019) [24]; Huang et al. (2008) [22]; Miettinen (2014) [17]; Vaquero et al. (2009) [23]; Vilos et al. (2012) [5]	119	27–68	Yes: 15, No: 104	Cellular atypia, necrosis, VEGF expression	Hysterectomy	6–60
Hakverdi et al. (2011) [9]; Ng et al. (2010) [16]; Zhang et al. (2014) [15]	87	26–66	Yes: 11, No: 76	Atypical leiomyoma, variable histology, molecular analysis	Hysterectomy, myomectomy	6–60

## 4. Conclusions

Diagnosing Smooth Muscle Tumors of Uncertain Malignant Potential (STUMPs) remains challenging due to the lack of standardized diagnostic criteria and the overlap in presentation with other smooth muscle tumors. As stated, “there is no standard diagnosis for the condition and its presentation overlaps with other conditions involving tumors in the smooth muscles making definitive classification difficult. Key characteristics that aid in identifying STUMPs include varying degrees of cytologic atypia, mitotic activity, and the presence or absence of coagulative necrosis.

Symptoms of STUMPs, such as the presence of abnormal bleeding, rapid growth, and pressure symptoms, overlap with those of leiomyomas, complicating the differentiation process further. Tumor sizes vary widely from 0.7 cm to 18 cm, depending on the stage, and tumors often display irregular and unpredictable growth patterns.

Key diagnostic criteria include those introduced by Dall’Asta et al. (2014). These criteria emphasize that STUMPs typically exhibit more pronounced cellular atypia than typical L, an elevated mitotic index that is below the threshold for malignancy, and minimal or absent necrosis and hemorrhage. While medical imaging tools such as ultrasound, MRI, and CT scans play a role in evaluating tumors, their ability to diagnose STUMPs specifically is limited. These modalities often show features suggestive of leiomyomas but cannot reliably differentiate STUMPs from benign tumors due to overlapping imaging characteristics. Ultrasound may reveal features indicative of L, while MRI and CT scans might show well-defined margins and homogeneous enhancement but lack distinctive features for STUMPs. Immunohistochemical staining is also a critical tool in diagnosing STUMPs.

Abdominal hysterectomy is the primary treatment for STUMPs and generally results in a low recurrence rate. Despite this, recurrences can occur and may be delayed, sometimes emerging several months after surgery. Factors such as histopathological aggressiveness and the extent of primary surgery play significant roles in the likelihood of recurrence. More targeted surgical approaches based on tumor characteristics might help reduce recurrence rates. Fertility-sparing approaches used for less advanced cases, such as tumorectomy or morcellation, may be considered. These methods preserve the uterus but carry risks, particularly if used without a definitive preoperative diagnosis or in cases of occult sarcomas. Close monitoring and follow-up are crucial for patients opting for these procedures. Hormonal substitution therapy after surgery may be recommended to manage symptoms and support overall reproductive health. This therapy can help alleviate menopausal symptoms and maintain hormonal balance, particularly for younger patients who wish to preserve their fertility.

The treatment of recurrence or metastasis of STUMP (Smooth Muscle Tumor of Uncertain Malignant Potential) can be challenging and must be individualized based on the specifics of each case. Each case of STUMP recurrence or metastasis should be discussed with a multidisciplinary team to determine the best course of action tailored to the patient’s specific condition and overall health.

The bivariate analysis revealed no statistically significant associations between recurrence risk and mitotic count, cytologic atypia, or coagulative necrosis in STUMP cases. Although some recurrences were observed, the overall rate was low (8.1%), indicating that, based on this sample, the statistical significance of recurrence risk in STUMPs is not high.

In summary, while STUMPs present diagnostic and treatment challenges, ongoing research and refined management strategies, including fertility-sparing and hormonal therapy options, offer hope for improving patient outcomes and addressing the complexities associated with these tumors.

## Data Availability

The data presented in this study are available on request from the corresponding author. The data are not publicly available due to internal rules.

## Supplementary Materials

The following supporting information can be downloaded at: https://www.mdpi.com/article/10.3390/curroncol31090388/s1, PRISMA checklist [31].
