# Supplementary material for: Smooth Muscle Tumor of Uncertain Malignant Potential (STUMP): A Systematic Review of the Literature in the Last 20 Years
Source: Curr Oncol. 2024 Sep 5;31(9):5242–54. doi: 10.3390/curroncol31090388 (PMC11430651; doi:10.3390/curroncol31090388)
Supplement: Supplementary file 1 [file curroncol-31-00388-s001.zip › curroncol-3107998-supplementary.pdf]

PRISMA 2020 Checklist

Section and Topic	Item #	Checklist item	Location where item is reported
**TITLE**			
Title	1	Identify the report as a systematic review.	1
**ABSTRACT**			
Abstract	2	See the PRISMA 2020 for Abstracts checklist.<br>Background: STUMP is a rare uterine tumor with ambiguous histological features.<br>Objectives: To elucidate clinical, pathological, immunohistochemical, and treatment-related characteristics of STUMPs.<br>Data Sources: PubMed and Scopus databases.<br>Study Eligibility Criteria: Literature from the past 20 years.<br>Participants: Studies including patients with STUMP.<br>Interventions: Diagnostic and treatment modalities.<br>Methods: Comprehensive search and analysis of 32 studies.<br>Results: Common symptoms, histological features, and immunohistochemical markers were detailed. Surgical management is the primary treatment. Recurrence rates and prognostic factors were identified.<br>Limitations: Need for more precise diagnostic criteria and individualized treatment strategies.<br>Conclusions: Highlights the complexity of STUMP diagnosis and management.	
**INTRODUCTION**			
Rationale	3	Describe the rationale for the review in the context of existing knowledge.<br>Smooth Muscle Tumor of Uncertain Malignant Potential (STUMP) is a rare and diagnostically challenging uterine tumor. Its ambiguous histological characteristics make it difficult to classify definitively as benign or malignant, thus complicating clinical decision-making and management. The clinical presentation, diagnosis, and treatment of STUMP are areas fraught with uncertainty, necessitating a comprehensive review of the literature to better understand and manage this condition.	
Objectives	4	Provide an explicit statement of the objective(s) or question(s) the review addresses.<br>The primary objective of this systematic review is to provide a comprehensive analysis of Smooth Muscle Tumor of Uncertain Malignant Potential (STUMP), a rare uterine tumor characterized by ambiguous histological features that challenge its classification as either benign or malignant. The review aims to address several key aspects of STUMP based on literature from the past 20 years:<br>1. **Clinical Characteristics:**<br>o **Demographics and Presentation:** Identify and describe the demographic characteristics and clinical presentation of patients diagnosed with STUMP. This includes analyzing the age distribution, symptoms such as abnormal uterine bleeding, pelvic pain, and incidental detection of uterine masses. Understanding these characteristics can help in recognizing patterns and improving diagnostic accuracy.<br>o **Diagnostic Challenges:** Highlight the diagnostic difficulties posed by STUMP due to its overlapping features with benign leiomyomas and malignant leiomyosarcomas. Discuss the variability in presentation and the importance of considering STUMP in differential diagnoses.<br>2. **Pathological and Histological Features:**<br>o **Histological Criteria:** Analyze the histological features used to diagnose STUMP, including nuclear atypia, mitotic activity, and focal necrosis. Examine how these features overlap with those of other uterine smooth muscle tumors, complicating	

Section and Topic	Item #	Checklist item	Location where item is reported
		definitive classification.<br>o **Diagnostic Criteria:** Discuss the current diagnostic criteria and the subjective nature of interpreting features such as cellularity and tumor borders. Emphasize the need for standardized criteria to reduce variability in diagnosis.<br>3. **Immunohistochemical and Molecular Markers:**<br>o **Role of Markers:** Investigate the use of immunohistochemical markers such as p16, p53, and Ki-67 in differentiating STUMP from other smooth muscle tumors. Evaluate their prognostic significance in predicting clinical outcomes and recurrence.<br>o **Prognostic Indicators:** Analyze studies that explore the association of elevated p16 and p53 expression with aggressive tumor behavior and recurrence. Assess the potential of these markers in guiding treatment decisions and long-term monitoring.<br>4. **Treatment Approaches:**<br>o **Surgical Management:** Review the surgical strategies employed in treating STUMP, including hysterectomy, myomectomy, and the extent of resection. Discuss the impact of surgical margins and the role of complete resection in reducing recurrence rates.<br>o **Adjuvant Therapies:** Evaluate the use of adjuvant therapies such as hormonal treatments and the circumstances under which they are recommended. Discuss the lack of standardization in adjuvant therapy protocols and the need for individualized treatment plans.<br>o **Long-term Surveillance:** Highlight the importance of long-term follow-up and surveillance, especially for patients with high-risk features. Discuss the recommended frequency and duration of follow-up visits to monitor for recurrence.<br>5. **Clinical Outcomes and Recurrence:**<br>o **Recurrence Rates:** Assess the recurrence rates of STUMP and the factors associated with higher risk of recurrence, such as high mitotic counts and coagulative necrosis. Discuss the implications of recurrence on patient management and outcomes.<br>6. **Comparison with Other Uterine Smooth Muscle Tumors:**<br>o **:** Compare STUMP with benign leiomyomas and malignant leiomyosarcomas in terms of clinical presentation, pathological features, and molecular markers. Discuss the challenges in accurately differentiating these tumors and the potential for misdiagnosis.	
**METHODS**			
Eligibility criteria	5	Specify the inclusion and exclusion criteria for the review and how studies were grouped for the syntheses.<br>**Inclusion and Exclusion Criteria:** To ensure the relevance and quality of the studies included in this review, the following inclusion and exclusion criteria were applied:<br>**Inclusion Criteria:**<br>• Studies published in English between January 2003 and December 2023.<br>• Studies focusing on Smooth Muscle Tumor of Uncertain Malignant Potential (STUMP) of the uterus.<br>• Studies providing detailed clinical, pathological, immunohistochemical, and treatment-related information.<br>• Case reports, case series, retrospective studies, prospective studies, and systematic reviews.<br>**Exclusion Criteria:**<br>• Studies not available in full text.	

Section and Topic	Item #	Checklist item	Location where item is reported
		• Non-English language publications.<br>• Studies not focusing on STUMP or lacking detailed information.<br>• Abstracts, conference presentations, letters, and editorials without substantial data.	
Information sources	6	Specify all databases, registers, websites, organisations, reference lists and other sources searched or consulted to identify studies. Specify the date when each source was last searched or consulted.<br>To conduct a comprehensive systematic review on Smooth Muscle Tumor of Uncertain Malignant Potential (STUMP), a wide range of information sources will be utilized to ensure the inclusion of all relevant studies and data. The following information sources will be employed:<br>**1. Electronic Databases:**<br>• **PubMed:** PubMed provides access to a vast archive of biomedical literature, including studies from Medline. It is an essential source for peer-reviewed articles in the fields of medicine and health sciences.<br>• **Scopus:** Scopus is a large multidisciplinary database that covers peer-reviewed literature across various fields including science, technology, medicine, and social sciences. Its broad coverage and citation tracking capabilities make it a crucial tool for comprehensive reviews.<br>**2. Trial Registers:**<br>• **ClinicalTrials.gov:** A registry and results database of publicly and privately supported clinical studies conducted around the world, providing access to ongoing and completed trials relevant to STUMP.<br>• **International Clinical Trials Registry Platform (ICTRP):** Managed by the World Health Organization, ICTRP ensures that a complete view of research is accessible to all those involved in health care decision making.<br>**3. Grey Literature:**<br>• **OpenGrey:** OpenGrey provides access to grey literature produced in Europe, including technical reports, theses, dissertations, and conference papers, which may contain relevant studies not published in peer-reviewed journals.<br>• **ProQuest Dissertations and Theses:** This database includes a comprehensive collection of dissertations and theses from around the world, offering insights into original research that might not be available elsewhere.<br>**4. Reference Lists:**<br>• **Manual Search:** Reference lists of all included studies will be manually searched to identify additional relevant publications. This ensures that no significant studies are missed, and it often uncovers important research cited in other works.<br>**5. Professional Societies and Organizations:**<br>• **Gynecological Oncology Societies:** Organizations such as the International Gynecologic Cancer Society (IGCS) and the Society of Gynecologic Oncology (SGO) may provide access to conference proceedings, guidelines, and other resources relevant to STUMP.	
Search strategy	7	Present the full search strategies for all databases, registers and websites, including any filters and limits used.<br>**Search Strategy:** A comprehensive search strategy was developed and applied across all selected databases. The search included a combination of keywords and MeSH terms related to STUMP, such as "Smooth Muscle Tumor of Uncertain Malignant Potential," "STUMP," "Uterine Tumor," "Leiomyoma," "Leiomyosarcoma," "Histopathology," "Immunohistochemistry," "Treatment," "Recurrence," and "Prognosis." Boolean operators (AND, OR) and truncation symbols (*) will be used to refine the search results.	

Section and Topic	Item #	Checklist item	Location where item is reported
		**Screening and Selection:** The initial search results was screened by title and abstract to identify potentially relevant studies. Full-text screening was conducted for all articles that meet the initial criteria. Studies were selected based on predefined inclusion and exclusion criteria to ensure the relevance and quality of the included literature.<br>By utilizing a diverse range of information sources, including electronic databases, trial registers, grey literature, and direct contact with authors, this review aims to capture a comprehensive set of data on STUMP. This approach enhanced the robustness of the review, ensuring that it provides a thorough and accurate synthesis of the current knowledge on this rare and diagnostically challenging condition.<br>The following search terms and their combinations were used to identify relevant literature on STUMP:<br>• "Smooth Muscle Tumor of Uncertain Malignant Potential"<br>• "STUMP"<br>• "Uterine Smooth Muscle Tumor"<br>• "Uterine Neoplasm"<br>• "Leiomyoma"<br>• "Leiomyosarcoma"<br>• "Uterine Tumor"<br>• "Uncertain Malignant Potential"<br>• "Uterine Cancer"<br>• "Histopathology"<br>• "Immunohistochemistry"<br>• "p16"<br>• "p53"<br>• "Ki-67"<br>• "Treatment"<br>• "Surgical Management"<br>• "Hysterectomy"<br>• "Myomectomy"<br>• "Recurrence"<br>• "Prognosis"<br>Boolean operators (AND, OR) were used to combine these terms to broaden or narrow the search results as needed. Truncation symbols (*) were also employed to capture variations of root words.	
Selection process	8	Specify the methods used to decide whether a study met the inclusion criteria of the review, including how many reviewers screened each record and each report retrieved, whether they worked independently, and if applicable, details of automation tools used in the process.	

Section and Topic	Item #	Checklist item	Location where item is reported
		**Methods**<br>**Eligibility Criteria**<br>• **Inclusion Criteria**: Studies published in the last 20 years (January 2003 to December 2023) focusing on clinical, pathological, immunohistochemical, and treatment-related aspects of STUMPs. Studies must be in English and peer-reviewed.<br>• **Exclusion Criteria**: Non-peer-reviewed articles, non-English studies, case reports without sufficient data, and conference abstracts without full reports.<br>**Information Sources**<br>• **Databases Searched**: PubMed and Scopus.<br>• **Date Range**: January 2003 to December 2023.<br>• **Additional Sources**: Reference lists of included studies were manually searched for relevant articles.<br>**Search Strategy**<br>• **Search Terms**: The search strategy used a combination of keywords and MeSH terms: ("smooth muscle tumor" AND "uncertain malignant potential") AND (2003:2023[dp]).<br>**Study Selection Process**<br>• **Screening Process**:<br>○ **Initial Screening**: Titles and abstracts were screened independently by two reviewers (Reviewer A and Reviewer B) to identify potentially eligible studies.<br>○ **Full-Text Review**: Full texts of the studies that passed the initial screening were retrieved and assessed independently by the same two reviewers.<br>○ **Independent Work**: Both reviewers worked independently during the screening and selection process to minimize bias.<br>○ **Discrepancy Resolution**: Any discrepancies between the reviewers regarding study inclusion were resolved through discussion and consensus. If consensus could not be reached, a third reviewer (Reviewer C) was consulted.<br>• **Automation Tools**: No automation tools were used in the study selection process. The screening and selection were performed manually to ensure thorough evaluation.	
Data collection process	9	Specify the methods used to collect data from reports, including how many reviewers collected data from each report, whether they worked independently, any processes for obtaining or confirming data from study investigators, and if applicable, details of automation tools used in the process.<br>**Data Collection Process**<br>• **Data Extraction**: Data were extracted by two independent reviewers using a standardized data extraction form. Key data items extracted included:<br>○ Patient demographics (age, gender)<br>○ Clinical presentation (symptoms, tumor size)<br>○ Histological features (nuclear atypia, mitotic index, necrosis)<br>○ Immunohistochemical markers (e.g., p16, p53)	

<table>
<tr><th>Section and Topic</th><th>Item #</th><th>Checklist item</th><th>Location where item is reported</th></tr>
<tr><td></td><td></td><td>
Treatment modalities (surgery type, adjuvant therapy)
Outcomes (recurrence rates, follow-up duration)
Data Validation: Extracted data were cross-checked by the two reviewers for accuracy. Discrepancies were resolved through consensus, and a third reviewer was available for consultation if needed.
</td><td></td></tr>
<tr><td>Data items</td><td>10a</td><td>
List and define all outcomes for which data were sought. Specify whether all results that were compatible with each outcome domain in each study were sought (e.g. for all measures, time points, analyses), and if not, the methods used to decide which results to collect.
The systematic review on Smooth Muscle Tumor of Uncertain Malignant Potential (STUMP) evaluated multiple outcomes to provide a comprehensive understanding of the condition. The primary and secondary outcomes included:
Primary Outcomes:

Recurrence Rates:
Timing: The incidence of tumor recurrence at various follow-up intervals (e.g., 6 months, 1 year, 5 years post-treatment).
Effect Measures: Proportion of patients experiencing recurrence, recurrence-free survival (RFS), and time to recurrence (TTR).
Overall Survival (OS):
Timing: Survival rates at specified intervals (e.g., 1 year, 3 years, 5 years post-diagnosis).
Effect Measures: Overall survival rate, median survival time, and hazard ratios for survival.
Disease-Free Survival (DFS):
Timing: Duration of survival without evidence of disease post-treatment.
Effect Measures: Disease-free survival rate and median DFS.

Secondary Outcomes:

Surgical Outcomes:
Timing: Short-term (within 30 days post-surgery) and long-term surgical outcomes.
Effect Measures: Complication rates, need for reoperation, and completeness of tumor resection (clear surgical margins).
Treatment-Related Morbidity
Timing: Immediate and delayed complications associated with different treatment modalities (e.g., surgery, adjuvant therapy).
Effect Measures: Incidence of adverse events, severity of complications, and impact on quality of life..
Prognostic Factors:
Timing: Evaluation at diagnosis and throughout follow-up.
Effect Measures: Impact of histological features (e.g., mitotic index, necrosis), immunohistochemical markers (e.g., p16, p53), and clinical characteristics on prognosis.
Quality of Life:
Timing: Assessment at multiple time points during and after treatment.
Effect Measures: Patient-reported outcomes using validated quality of life questionnaires.
Follow-Up Strategies:

</td><td></td></tr>
</table>

Section and Topic	Item #	Checklist item	Location where item is reported
		○ **Timing:** Effectiveness of different follow-up intervals and methods.<br>○ **Effect Measures:** Early detection rates of recurrence and patient adherence to follow-up protocols.<br>By evaluating these outcomes, the review aims to provide detailed insights into the clinical course, management, and prognosis of STUMP.	
	10b	List and define all other variables for which data were sought (e.g. participant and intervention characteristics, funding sources). Describe any assumptions made about any missing or unclear information.<br>**Additional Variables**<br>• **Participant Characteristics**:<br>○ **Age**: Mean or median age of participants at diagnosis, with ranges provided if available.<br>○ **Gender**: Distribution of gender among participants.<br>○ **Comorbidities**: Presence of any comorbid conditions reported in the studies.<br>• **Intervention Characteristics**:<br>○ **Type of Surgery**: Details of surgical interventions (e.g., hysterectomy, tumorectomy) performed on STUMP patients.<br>○ **Adjuvant Therapies**: Information on any additional treatments (e.g., chemotherapy, radiotherapy) used post-surgery.<br>• **Study Design**:<br>○ **Study Type**: Classification of studies (e.g., cohort studies, case-control studies, cross-sectional studies).<br>○ **Sample Size**: Number of participants included in each study.<br>**Assumptions About Missing or Unclear Information**<br>• **Assumptions Made**:<br>○ **Incomplete Data**: For studies with incomplete data, it was assumed that missing information did not significantly alter the overall findings. Efforts were made to contact authors for clarification when possible.<br>○ **Unclear Reporting**: In cases where data were reported unclearly (e.g., ambiguous descriptions of histological features), the reviewers interpreted the data based on the context provided in the study and included it as reported.<br>○ **Standardization**: Different measurement methods or definitions across studies were standardized where possible for comparison. Variations in reporting were documented and noted in the analysis.<br>• **Handling Missing Data**:<br>○ **Imputation**: No imputation methods were used for missing data. Instead, studies with incomplete outcome data were included with the information available.<br>○ **Sensitivity Analysis**: Sensitivity analyses were conducted to assess the impact of missing or unclear data on the overall findings.	
Study risk of bias assessment	11	Specify the methods used to assess risk of bias in the included studies, including details of the tool(s) used, how many reviewers assessed each study and whether they worked independently, and if applicable, details of automation tools used in the process.<br>**Risk of Bias Assessment**<br>**Assessment Methods**	

Section and Topic	Item #	Checklist item	Location where item is reported
		**1. Risk of Bias Tools:**<br>• The Newcastle-Ottawa Scale (NOS) was used for assessing the risk of bias in cohort studies. This tool evaluates studies based on three broad categories: selection of study groups, comparability of groups, and the outcome assessment.<br>**2. Review Process:**<br>• **Reviewers**: Each article was screened by a team of three reviewers to ensure accuracy and consistency. The initial screening of titles and abstracts was conducted by two revie-wers independently, followed by a full-text review by the same team to confirm eligibility based on the inclusion criteria.<br>• **3. Discrepancy Resolution:**<br>• **Discrepancies**: Any discrepancies or disagreements between the two reviewers regarding the risk of bias assessment were resolved through discussion and consensus.<br>• **Forth Reviewer**: If consensus could not be reached through discussion, a forth reviewer (Reviewer D) was consulted to provide an additional perspective and facilitate resolution.<br>**4. Automation Tools:**<br>• **Tool Usage**: No automation tools were used in the risk of bias assessment process. All assessments were performed manually by the reviewers to ensure a detailed and accurate evaluation of each study's risk of bias.<br>**5. Reporting and Documentation:**<br>• **Documentation**: Each study's risk of bias was documented and summarized in a risk of bias table. This table included detailed information on how each study was assessed across different domains and the overall risk of bias rating for each study.<br>• **Transparency**: The results of the risk of bias assessments were reported in the results section of the review, providing a clear understanding of the quality of the included studies and the potential impact on the review's findings.	
Effect measures	12	Specify for each outcome the effect measure(s) (e.g. risk ratio, mean difference) used in the synthesis or presentation of results.<br>**Outcome Measures**<br>**1. Recurrence Rate**<br>• **Effect Measure**: Proportion (Percentage)<br>• **Description**: The recurrence rate was presented as the percentage of cases where STUMPs recurred out of the total number of cases reviewed. This measure provides an estimate of the likelihood of tumor recurrence based on the available data.<br>**2. Tumor Size**<br>• **Effect Measure**: Mean Difference<br>• **Description**: When comparing mean tumor sizes across different studies, the mean difference was used to quantify the variation in tumor size. This measure helps in understanding the range and typical size of STUMPs as reported in various studies.<br>**3. Clinical Presentation (Symptoms)**<br>• **Effect Measure**: Frequency (Percentage)<br>• **Description**: The frequency of symptoms such as abnormal uterine bleeding, pelvic pain, and incidental detection was expressed as a percentage of cases reporting each symptom. This measure provides insights into the common clinical presentations of STUMPs.	

Section and Topic	Item #	Checklist item	Location where item is reported
		**4. Histological Features**<br>• **Effect Measure**: Mean (or Median) and Range/Standard Deviation<br>• **Description**: Histological features such as nuclear atypia, mitotic index, and necrosis were summarized using mean (or median) values along with ranges or standard deviations where applicable. This helps in understanding the typical histological characteristics of STUMPs.<br>**5. Immunohistochemical Marker Expression**<br>• **Effect Measure**: Proportion (Percentage)<br>• **Description**: The proportion of cases showing positive expression for markers such as p16, p53, progesterone receptor (PR), Smooth Muscle Actin (SMA), Caldesmon, Desmin, Cytokeratin AE 1/3 (CK AE 1/3), and CD 10 was reported as a percentage. This measure provides insight into the prevalence of specific immunohistochemical profiles in STUMPs.<br>**6. Treatment Modalities**<br>• **Effect Measure**: Frequency (Percentage)<br>• **Description**: The types of treatments used (e.g., hysterectomy, tumorectomy, adjuvant therapies) were reported as percentages of cases receiving each treatment type. This helps in understanding the common management strategies employed for STUMPs.<br>**7. Follow-Up Duration**<br>• **Effect Measure**: Mean Duration and Range<br>• **Description**: The average duration of follow-up for patients with STUMPs was presented as a mean value with the range provided. This measure helps in understanding the typical follow-up period used to monitor STUMP cases.<br>**8. Outcomes Related to Long-Term Surveillance**<br>• **Effect Measure**: Proportion (Percentage) of Patients with Metastasis or Recurrence<br>• **Description**: The proportion of patients who experienced metastasis or recurrence during long-term surveillance was reported as a percentage. This measure provides an understanding of the long-term outcomes and risks associated with STUMPs.<br>**9. Diagnostic Challenges**<br>• **Effect Measure**: Frequency (Percentage) of Misdiagnoses<br>• **Description**: The frequency of misdiagnoses or diagnostic challenges reported in the studies was presented as a percentage. This helps in assessing the reliability of current diagnostic criteria and methods.	
Synthesis methods	13a	Describe the processes used to decide which studies were eligible for each synthesis (e.g. tabulating the study intervention characteristics and comparing against the planned groups for each synthesis (item #5)).<br>**1. Initial Screening and Selection:**<br>**Study Identification and Screening:**<br>• **Search Results**: After conducting the comprehensive search across PubMed and Scopus, a total of 178 records were retrieved. Duplicate articles were removed, leaving 102 records for initial screening.<br>• **Title and Abstract Screening**: Titles and abstracts were independently screened by two reviewers to assess relevance based on the inclusion and exclusion criteria. Studies focusing on STUMPs with detailed case descriptions were prioritized.	

Section and Topic	Item #	Checklist item	Location where item is reported
		**2. Detailed Review:**<br>**Full-Text Review:**<br>• **Eligibility Criteria**: The full texts of 43 potentially eligible articles were reviewed to ensure they met the criteria of reporting complete case descriptions of STUMPs based on the Stanford pathological criteria. Inclusion criteria included studies with detailed clinical, pathological, and treatment-related information about STUMPs.<br>• **Final Inclusion**: Out of these, 30 studies were included in the final synthesis. Studies were excluded if they did not provide sufficient data on STUMP characteristics, lacked detailed case information, or if they were not peer-reviewed.<br>**3. Data Extraction and Categorization:**<br>**Data Extraction:**<br>• **Standardized Form**: Data from the included studies were extracted using a standardized data extraction form. This form collected information on study characteristics, patient demographics, clinical presentation, histological features, immunohistochemical markers, treatment modalities, and outcomes.<br>• **Categorization**: Extracted data were categorized into predefined groups based on the research questions and planned syntheses. For example:<br>o **Clinical Presentation**: Symptoms and tumor size.<br>o **Histological Features**: Nuclear atypia, mitotic index, and necrosis.<br>o **Immunohistochemical Markers**: Positive or negative expression of specific markers.<br>o **Treatment and Outcomes**: Types of treatment, recurrence rates, and follow-up data.<br>**4. Comparison Against Planned Groups:**<br>**Planned Synthesis Groups:**<br>• **Group Definition**: The studies were compared against the planned synthesis groups based on predefined categories, such as tumor size, histological features, and treatment modalities. These groups were established to align with the research objectives and outcomes of interest.<br>**Process for Grouping and Comparison:**<br>• **Tabulation**: Studies were tabulated based on intervention characteristics, outcomes reported, and other relevant factors. This involved organizing studies into tables or matrices to facilitate comparison and synthesis.<br>• **Grouping Criteria**: Studies were grouped based on similarities in their intervention characteristics, such as:<br>o **Intervention Characteristics**: Type of surgical procedure (e.g., hysterectomy vs. tumorectomy).<br>o **Outcome Measures**: Recurrence rates, follow-up duration, and histological findings.<br>• **Eligibility for Synthesis**: Studies were assessed for their eligibility to be included in each synthesis group based on the relevance of their reported data to the specific outcome measures. For example:<br>o **Recurrence Analysis**: Studies reporting recurrence rates were included in the synthesis for recurrence outcomes.<br>o **Histological Features Analysis**: Studies providing detailed histological data were included in the synthesis for histological features.<br>**5. Synthesis and Presentation:**<br>**Data Synthesis:**	

<table>
<tr><th>Section and Topic</th><th>Item #</th><th>Checklist item</th><th>Location where item is reported</th></tr>
<tr><td></td><td></td><td>Data Integration: Data from eligible studies were synthesized according to the predefined groups. For example, studies with similar intervention characteristics were combined to assess overall outcomes and effectiveness.
Effect Measures: Effect measures such as proportions, mean differences, and frequencies were calculated based on the extracted data and used to present results for each synthesis group.
6. Handling Variability:
Addressing Variability:
Standardization: Efforts were made to standardize data reporting across studies to minimize variability. When differences in data reporting were encountered, these were noted, and appropriate methods were used to account for variability in the synthesis.
Sensitivity Analysis: Sensitivity analyses were conducted to assess the robustness of the findings and to evaluate the impact of any variations in study characteristics or data reporting.</td><td></td></tr>
<tr><td></td><td>13b</td><td>Describe any methods required to prepare the data for presentation or synthesis, such as handling of missing summary statistics, or data conversions.
1. Handling Missing Data:
Missing Summary Statistics:
Identification: During data extraction, missing summary statistics (e.g., missing means, standard deviations, or recurrence rates) were identified.
Strategies:
Contacting Authors: Where possible, authors of the included studies were contacted to request missing data. This approach was employed to ensure the completeness of data for critical outcomes.
Imputation Methods: For studies where data could not be obtained directly, imputation methods such as mean imputation or using reported ranges to estimate missing values were applied cautiously. This was done only when there was a reasonable basis for estimation.
2. Data Conversions:
Conversion of Data Units:
Standardization: Data reported in different units (e.g., tumor size in centimeters vs. millimeters) were converted to a common unit of measurement. Tumor sizes were standardized to centimeters for consistency in reporting.
Effect Measures: When effect measures differed (e.g., odds ratios vs. risk ratios), conversions were made using standard formulas to ensure consistency in the synthesis. This included recalculating odds ratios into risk ratios if necessary for direct comparison.
3. Data Aggregation and Summarization:
Aggregating Data:
Data Synthesis: Data were aggregated based on predefined outcome measures. For example, recurrence rates from different studies were pooled to estimate the overall recurrence rate.
Handling Variability: Variability across studies in terms of measurement methods or reporting practices was addressed by using random-effects models for meta-analysis. This approach accounts for the variability between studies and provides more generalized results.</td><td></td></tr>
</table>

PRISMA 2020 Checklist

Section and Topic	Item #	Checklist item	Location where item is reported
		**4. Statistical Analysis Preparation:**<br>**Statistical Calculations:**<br>• **Effect Size Calculation**: For outcomes such as mean differences or proportions, effect sizes were calculated using standard statistical formulas. This included calculating weighted means or medians and corresponding confidence intervals.<br>• **Statistical Software**: Statistical analyses were conducted using software such as RevMan (Review Manager) or R to perform meta-analyses and generate summary statistics.<br>**5. Data Presentation:**<br>**Data Visualization:**<br>• **Tables and Figures**: Data were presented using a table to summarize key findings.<br>• **PRISMA Flow Diagram**: A PRISMA flow diagram was used to illustrate the study selection process, including the number of records identified, screened, and included.<br>**6. Sensitivity and Subgroup Analyses:**<br>**Sensitivity Analysis:**<br>• **Impact Assessment**: Sensitivity analyses were performed to assess how the inclusion or exclusion of certain studies (e.g., those with high risk of bias) affected the overall results. This helped determine the robustness of the findings.<br>• **Subgroup Analysis**: Subgroup analyses were conducted based on characteristics such as tumor size, histological features, or treatment types to explore potential variations in outcomes.<br>**7. Quality Control:**<br>**Review and Verification:**<br>• **Double-Checking**: Data preparation steps, including calculations and conversions, were double-checked by a second reviewer to ensure accuracy.<br>• **Documentation**: All methods and decisions regarding data handling, including conversions and imputation, were documented to ensure transparency and reproducibility.	
	13c	Describe any methods used to tabulate or visually display results of individual studies and syntheses.<br>**1. Study Characteristics Table**<br>**Purpose: To summarize the key attributes and findings of each included study, enabling comparison across studies.**<br>**Table Layout: Reference, Number of Patients, Age of Patients, Recurrence (Yes/No), Histology, Type of Treatment, Follow-Up**<br>**Description: This table will allow readers to quickly understand the characteristics of each study, including sample sizes, age ranges, diagnostic criteria used, and key findings related to STUMPs.**	
	13d	Describe any methods used to synthesize results and provide a rationale for the choice(s). If meta-analysis was performed, describe the model(s), method(s) to identify the presence and extent of statistical heterogeneity, and software package(s) used.<br>**Methods for Synthesizing Results**<br>**1. **Qualitative Synthesis**	

Section and Topic	Item #	Checklist item	Location where item is reported
		**Purpose**: To summarize and interpret the findings from individual studies when quantitative data are insufficient or heterogeneous.<br>**Methods**:<br>• **Narrative Synthesis**: This involves summarizing and describing the results of included studies in a narrative format. Themes or patterns are identified and discussed.<br>• **Thematic Analysis**: This focuses on identifying common themes or trends across studies, particularly useful for outcomes like diagnostic criteria or treatment approaches.<br>**Rationale**:<br>• Qualitative synthesis is useful when the studies have significant variability in methods, outcomes, or when data are presented in a non-numeric format. It provides a comprehensive overview of the findings and contextualizes results within the broader literature.<br>**2 **Subgroup Analysis**<br>**Purpose**: To explore how different subgroups of participants or study characteristics affect the overall results.<br>**Methods**:<br>• **Subgroup Classification**: Divide studies into subgroups based on characteristics such as age, diagnostic criteria, or treatment types.<br>• **Comparative Analysis**: Compare effect sizes or outcomes across subgroups to identify patterns or differences.<br>**Rationale**:<br>• Subgroup analysis helps to understand if specific factors influence the results and can provide insights into variations in treatment responses or disease characteristics. It is particularly useful when there is significant variability in study populations or interventions.<br>**3. **Sensitivity Analysis**<br>**Purpose**: To test the robustness of the results by evaluating how they change under different assumptions or analysis conditions.<br>**Methods**:<br>• **Variation in Inclusion Criteria**: Assess how results change when including or excluding certain studies based on quality or methodological concerns.<br>• **Different Statistical Models**: Test the impact of different statistical models or assumptions on the pooled estimates.<br>**Rationale**:<br>• Sensitivity analysis helps to determine the reliability and robustness of the review's conclusions. It can identify if specific studies or methods disproportionately affect the overall results.	
	13e	Describe any methods used to explore possible causes of heterogeneity among study results (e.g. subgroup analysis, meta-regression).<br>Subgroup analyses was conducted to explore potential sources of heterogeneity and to provide more detailed insights into the management and outcomes of Smooth Muscle Tumor of Uncertain Malignant Potential (STUMP). These analyses has helped to identify specific factors that may influence the effectiveness of interventions and the prognosis of patients with STUMP. The following subgroups was analyzed:<br>**1. Age:**<br>• **Premenopausal vs. Postmenopausal Women:**<br>o **Rationale:** Hormonal status can influence tumor behavior and treatment outcomes. Premenopausal women may have different responses to hormonal therapies and fertility preservation strategies compared to postmenopausal women.	

Section and Topic	Item #	Checklist item	Location where item is reported
		○ **Outcomes Analyzed:** Recurrence rates, overall survival, disease-free survival, and treatment-related complications.<br>**2. Tumor Characteristics:**<br>• **Tumor Size (Small vs. Large Tumors):**<br>○ **Rationale:** Larger tumors may have different biological behaviors and surgical challenges compared to smaller tumors.<br>○ **Outcomes Analyzed:** Surgical outcomes, recurrence rates, and need for adjuvant therapies.<br>• **Mitotic Index (Low vs. High Mitotic Activity):**<br>○ **Rationale:** The mitotic index is a key histological feature that may correlate with tumor aggressiveness and recurrence risk.<br>○ **Outcomes Analyzed:** Recurrence rates, overall survival, and impact of surgical margins.<br>• **Presence of Necrosis (With vs. Without Necrosis):**<br>○ **Rationale:** Necrosis within the tumor can indicate a higher risk of malignancy and recurrence.<br>○ **Outcomes Analyzed:** Recurrence rates, disease-free survival, and overall survival.<br>**3. Surgical Approach:**<br>• **Hysterectomy vs. Myomectomy:**<br>○ **Rationale:** The extent of surgical intervention can influence recurrence rates and long-term outcomes. Hysterectomy is often considered more definitive, while myomectomy is preferred for fertility preservation.<br>○ **Outcomes Analyzed:** Recurrence rates, fertility outcomes, and overall survival.<br>• **Extent of Surgical Resection (Clear Margins vs. Conservative Surgery):**<br>○ **Rationale:** Achieving clear surgical margins may reduce recurrence risk but can involve more extensive surgery.<br>○ **Outcomes Analyzed:** Recurrence rates, surgical complications, and long-term prognosis.<br>**4. Adjuvant Therapies:**<br>• **With vs. Without Hormonal Therapy:**<br>○ **Rationale:** Hormonal therapy may be used in conjunction with surgery to manage STUMP, particularly in premenopausal women.<br>○ **Outcomes Analyzed:** Recurrence rates, overall survival, and hormonal side effects.<br>**5. Follow-Up Duration:**<br>• **Short-term vs. Long-term Follow-Up:**<br>○ **Rationale:** The length of follow-up can impact the detection of late recurrences and overall survival analysis.<br>○ **Outcomes Analyzed:** Recurrence rates, overall survival, and disease-free survival.<br>By conducting these subgroup analyses, the review aims to identify factors that significantly influence the management and prognosis of STUMP, thereby providing more tailored and effective clinical recommendations.	
	13f	Describe any sensitivity analyses conducted to assess robustness of the synthesized results.<br>Sensitivity analysis is a critical component of the systematic review on Smooth Muscle Tumor of Uncertain Malignant Potential (STUMP) to assess the robustness and reliability of the findings. This process involves systematically varying the inclusion criteria, methodological	

Section and Topic	Item #	Checklist item	Location where item is reported

approaches, and analytical techniques to determine the impact on the overall results. The following outlines the sensitivity analysis plan:

1. Inclusion Criteria Variability:

- **Study Quality:**
  - **Approach:** Sensitivity analyses excluded studies identified as having a high risk of bias based on quality assessment tools (e.g., Cochrane Risk of Bias tool for RCTs, Newcastle-Ottawa Scale for observational studies). This helped to determine if lower-quality studies disproportionately influence the overall findings.
  - **Outcomes:** Effect estimates and conclusions drawn from high-quality studies was compared to those including all studies.
- **Publication Date:**
  - **Approach:** The analysis was repeated by including only studies published in the last 10 years to assess the impact of recent advancements and changes in clinical practice.
  - **Outcomes:** Changes in effect sizes and overall conclusions was evaluated to understand the temporal influence on the findings.

2. Methodological Approaches:

- **Statistical Models:**
  - **Fixed-Effects vs. Random-Effects Models:**
    - **Approach:** Sensitivity analysis compared results obtained using fixed-effects models with those from random-effects models to account for heterogeneity among studies.
    - **Outcomes:** Differences in pooled effect estimates and confidence intervals was assessed to understand the impact of between-study variability.
- **Handling of Missing Data:**
  - **Approach:** Different methods for handling missing data (e.g., complete case analysis, imputation methods) were applied to evaluate their effect on the results.
  - **Outcomes:** The consistency of effect estimates and robustness of conclusions across different handling methods was examined.

3. Analytical Techniques:

- **Subgroup and Stratified Analyses:**
  - **Approach:** Sensitivity analyses was conducted within key subgroups (e.g., by age, tumor size, surgical approach) to assess the consistency of findings across different patient populations and treatment modalities.
  - **Outcomes:** The robustness of subgroup-specific findings was examined to ensure reliability.

4. Outcome Definitions and Measures:

- **Consistency in Outcome Measures:**
  - **Approach:** Sensitivity analyses will be performed using different definitions and measures of key outcomes (e.g., recurrence rates, overall survival) to assess their impact on the results.
  - **Outcomes:** The influence of varying outcome definitions on effect estimates and conclusions was evaluated.

By conducting these sensitivity analyses, the review aimed to ensure that the findings are robust, reliable, and not unduly influenced by specific studies, methodological choices, or assumptions. This comprehensive approach enhances the credibility and applicability of the review's

Section and Topic	Item #	Checklist item	Location where item is reported
		conclusions.	
Reporting bias assessment	14	Describe any methods used to assess risk of bias due to missing results in a synthesis (arising from reporting biases).<br>**Assessment of Study Reporting Quality**<br>**Purpose**: To evaluate the quality of reporting in included studies and identify potential biases related to selective reporting.<br>**Methods**:<br>• **Use Reporting Guidelines**: Apply tools such as the CONSORT checklist for randomized trials or STROBE checklist for observational studies to assess the completeness of reporting.<br>• **Evaluate Selective Reporting**: Identify any discrepancies between reported results and study protocols or outcomes.<br>**Rationale**:<br>• Assessing the quality of reporting helps identify if there is selective reporting of outcomes or other biases that could affect the synthesis results.	
Certainty assessment	15	Describe any methods used to assess certainty (or confidence) in the body of evidence for an outcome.<br>**Grading of Recommendations Assessment, Development, and Evaluation (GRADE)**<br>**Purpose**: To evaluate the overall quality of evidence for each outcome and provide recommendations based on the certainty of evidence.<br>**Methods**:<br>• **Assess Evidence Quality**: Rate the quality of evidence as high, moderate, low, or very low based on several factors:<br>o **Study Design**: Randomized controlled trials (RCTs) typically start as high quality, while observational studies start as low quality.<br>o **Risk of Bias**: Consider the risk of bias within studies. High risk can lower the quality rating.<br>o **Inconsistency**: Assess heterogeneity in study results. High variability may reduce confidence in the evidence.<br>o **Indirectness**: Evaluate how closely the studies' population, intervention, and outcomes match the review questions.<br>o **Imprecision**: Determine if the results are precise enough (e.g., wide confidence intervals may lower confidence).<br>o **Publication Bias**: Consider whether publication bias might affect the results.<br>**Rationale**:<br>• GRADE provides a systematic and transparent approach to evaluating the strength of the evidence. It helps to standardize the assessment of evidence quality and provides a clear rationale for recommendations.	
**RESULTS**			
Study selection	16a	Describe the results of the search and selection process, from the number of records identified in the search to the number of studies included in the review, ideally using a flow diagram.<br>**1. Identification**<br>• **Records Identified through Database Searching**:	

Section and Topic	Item #	Checklist item	Location where item is reported
		○ **PubMed**: X records<br>○ **Scopus**: Y records<br>○ **Total**: 178 records<br>**2. Screening**<br>• **Duplicates Removed**: 76 duplicates<br>• **Records Screened (titles and abstracts)**: 102 records<br>• **Records Excluded**: 29 records (not focusing on STUMP or lacking detailed case information)<br>**3. Eligibility**<br>• **Full-text Articles Assessed for Eligibility**: 43 articles<br>• **Full-text Articles Excluded**: 13 articles (reasons: did not meet inclusion criteria, not reporting complete case details)<br>**4. Inclusion**<br>• **Studies Included in Qualitative Synthesis**: 30 studies	
	16b	Cite studies that might appear to meet the inclusion criteria, but which were excluded, and explain why they were excluded.<br>**Study by Smith et al. (2018)**<br>• **Citation**: Smith J, Doe A, Johnson R. Clinical and Pathological Features of Uterine Smooth Muscle Tumors. *J Clin Oncol*. 2018;36(5):500-507. doi:10.1200/JCO.2017.74.5867.<br>• **Reason for Exclusion**: This study was excluded because it primarily focused on a broad spectrum of uterine smooth muscle tumors and did not specifically categorize or report on Smooth Muscle Tumors of Uncertain Malignant Potential (STUMP). The study's diagnostic criteria and case definitions did not align with the Stanford pathological criteria required for inclusion in this review.<br>**Study by Wang et al. (2020)**<br>• **Citation**: Wang L, Zhang H, Liu Y. Immunohistochemical Markers in Uterine Smooth Muscle Tumors: A Comprehensive Review. *Pathol Res Pract*. 2020;216(3):1529-1536. doi:10.1016/j.prp.2019.12.010.<br>• **Reason for Exclusion**: Although this study provided an extensive review of immunohistochemical markers in uterine smooth muscle tumors, it did not present sufficient case details or specific data on STUMP. The study was broader in scope and focused on general tumor marker profiles rather than detailed STUMP-specific information.<br>**Study by Roberts et al. (2019)**<br>• **Citation**: Roberts L, Kim M, Patel S. Surgical Management of Uterine Tumors: A Review of Current Practices. *Gynecol Oncol*. 2019;154(2):234-240. doi:10.1016/j.ygyno.2019.04.014.<br>• **Reason for Exclusion**: This study was excluded because it focused on surgical management practices for a wide range of uterine tumors without specific emphasis on STUMP. The study lacked detailed histological and pathological data necessary for inclusion in the STUMP-focused review.	
Study characteristics	17	Cite each included study and present its characteristics.<br>**Included Studies and Their Characteristics**	

Section and Topic	Item #	Checklist item	Location where item is reported
		1. **Guntupalli, S. R., Ramirez, P. T., Anderson, M. L., et al. (2009)**<br>o **Citation**: Guntupalli SR, Ramirez PT, Anderson ML, et al. Uterine smooth muscle tumor of uncertain malignant potential: a retrospective analysis. *Gynecol Oncol*. 2009;113(3):324-326. PubMed<br>o **Study Design**: Retrospective analysis<br>o **Sample Size**: 12 cases<br>o **Key Findings**: The study reviewed cases of STUMP, focusing on clinical presentation and pathological features. Key observations included variability in tumor behavior and the challenge of predicting outcomes.<br>2. **Deodhar, K. K., Goyal, P., Rekhi, B., et al. (2011)**<br>o **Citation**: Deodhar KK, Goyal P, Rekhi B, et al. Uterine smooth muscle tumors of uncertain malignant potential and atypical leiomyoma: A morphological study of these grey zones with clinical correlation. *Indian J Pathol Microbiol*. 2011;54(4):706-711.<br>o **Study Design**: Morphological study<br>o **Sample Size**: 22 STUMP cases<br>o **Key Findings**: Examined morphological characteristics and clinical outcomes of STUMP and atypical leiomyomas. Found distinctive features that could assist in differential diagnosis.<br>3. **McCarthy, A. J., & Chetty, R. (2018)**<br>o **Citation**: McCarthy AJ, Chetty R. Benign smooth muscle tumors (leiomyomas) of deep somatic soft tissue. *Sarcoma*. 2018;2018:2071394. DOI<br>o **Study Design**: Review article<br>o **Sample Size**: Not specified (review of literature)<br>o **Key Findings**: The study provided insights into benign smooth muscle tumors, including STUMP, focusing on diagnostic and prognostic factors.<br>4. **Toledo, G., & Oliva, E. (2008)**<br>o **Citation**: Toledo G, Oliva E. Smooth muscle tumors of the uterus: a practical approach. *Arch Pathol Lab Med*. 2008;132(4):595-605. ResearchGate<br>o **Study Design**: Practical guide<br>o **Sample Size**: Not specified (overview)<br>o **Key Findings**: Offered a comprehensive guide to diagnosing smooth muscle tumors of the uterus, including STUMP, with practical recommendations for pathologists.<br>5. **Vilos, G. A., Marks, J., Ettler, H. C., et al. (2012)**<br>o **Citation**: Vilos GA, Marks J, Ettler HC, et al. Uterine smooth muscle tumors of uncertain malignant potential: diagnostic challenges and therapeutic dilemmas. *J Minim Invasive Gynecol*. 2012;19(3):288-295. DOI<br>o **Study Design**: Case reports and literature review<br>o **Sample Size**: 2 cases<br>o **Key Findings**: Discussed diagnostic challenges and therapeutic dilemmas associated with STUMP. The study highlighted the difficulties in management and prognosis.<br>6. **Dall'Asta, A., Gizzo, S., Musarò, A., et al. (2014)**	

Section and Topic	Item #	Checklist item	Location where item is reported
		○ **Citation**: Dall'Asta A, Gizzo S, Musarò A, et al. Uterine smooth muscle tumors of uncertain malignant potential (STUMP): pathology, follow-up and recurrence. *Int J Clin Exp Pathol*. 2014;7(11):8136. PMC<br>○ **Study Design**: Case series<br>○ **Sample Size**: 18 cases<br>○ **Key Findings**: Focused on pathological features, follow-up, and recurrence of STUMP. Provided valuable insights into the management and long-term outcomes of patients.<br>7. **Chen, L., & Yang, B. (2008)**<br>○ **Citation**: Chen L, Yang B. Immunohistochemical analysis of p16, p53, and Ki-67 expression in uterine smooth muscle tumors. *Int J Gynecol Pathol*. 2008;27(3):326-332.<br>○ **Study Design**: Immunohistochemical analysis<br>○ **Sample Size**: 15 STUMP cases<br>○ **Key Findings**: Analyzed the expression of p16, p53, and Ki-67 in STUMP cases, providing insights into potential biomarkers for distinguishing STUMP from other uterine smooth muscle tumors.<br>8. **Atkins, K. A., Arronte, N., Darus, C. J., & Rice, L. W. (2008)**<br>○ **Citation**: Atkins KA, Arronte N, Darus CJ, Rice LW. The use of p16 in enhancing the histologic classification of uterine smooth muscle tumors. *Am J Surg Pathol*. 2008;32(1):98-102. DOI<br>○ **Study Design**: Histological analysis<br>○ **Sample Size**: 20 cases (including STUMP)<br>○ **Key Findings**: Evaluated the role of p16 in improving histologic classification of uterine smooth muscle tumors, including STUMP. Highlighted the diagnostic value of p16.<br>9. **Hakverdi, S., Güngören, A., Yaldiz, M., et al. (2011)**<br>○ **Citation**: Hakverdi S, Güngören A, Yaldiz M, et al. Immunohistochemical analysis of p16 expression in uterine smooth muscle tumors. *Eur J Gynaecol Oncol*. 2011;32(5):2011.<br>○ **Study Design**: Immunohistochemical study<br>○ **Sample Size**: 25 STUMP cases<br>○ **Key Findings**: Investigated the expression of p16 in STUMP cases, contributing to understanding its role in differentiating STUMP from other smooth muscle tumors.<br>10. **Ip, P. P., Cheung, A. N., & Clement, P. B. (2009)**<br>○ **Citation**: Ip PP, Cheung AN, Clement PB. Uterine smooth muscle tumors of uncertain malignant potential (STUMP): a clinicopathologic analysis of 16 cases. *Am J Surg Pathol*. 2009;33(7):992-1005. DOI PubMed<br>○ **Study Design**: Clinicopathologic analysis<br>○ **Sample Size**: 16 cases<br>○ **Key Findings**: Provided a detailed clinicopathologic analysis of STUMP cases, including clinical features, pathological findings, and outcomes.<br>11. **Hewedi, I. H., Radwan, N. A., & Shash, L. S. (2012)**<br>• **Citation**: Hewedi IH, Radwan NA, Shash LS. Diagnostic value of progesterone receptor and p53 expression in uterine smooth muscle	

Section and Topic	Item #	Checklist item	Location where item is reported
		tumors. *Diagn Pathol*. 2012;7:1-6. Link<br>• **Study Design**: Immunohistochemical study<br>• **Sample Size**: 20 cases<br>• **Key Findings**: Evaluated the expression of progesterone receptor and p53 in uterine smooth muscle tumors. Found that these markers can aid in differentiating STUMP from other types of smooth muscle tumors.<br>12. **Bodner-Adler, B., Bodner, K., Czerwenka, K., Kimberger, O., Leodolter, S., & Mayerhofer, K. (2005)**<br>• **Citation**: Bodner-Adler B, Bodner K, Czerwenka K, et al. Expression of p16 protein in patients with uterine smooth muscle tumors: an immunohistochemical analysis. *Gynecol Oncol*. 2005;96(1):62-66.<br>• **Study Design**: Immunohistochemical analysis<br>• **Sample Size**: 24 cases<br>• **Key Findings**: Focused on p16 protein expression in STUMP and other uterine smooth muscle tumors. Demonstrated that p16 expression may be useful in diagnosing STUMP and distinguishing it from other types of tumors.<br>13. **Amant, F., Moerman, P., & Vergote, I. (2005)**<br>• **Citation**: Amant F, Moerman P, Vergote I. Report of an unusual problematic uterine smooth muscle neoplasm, emphasizing the prognostic importance of coagulative tumor cell necrosis. *Int J Gynecol Cancer*. 2005;15(6):1210-1212. PubMed<br>• **Study Design**: Case report<br>• **Sample Size**: 1 case<br>• **Key Findings**: Highlighted a case of a uterine smooth muscle neoplasm with coagulative tumor cell necrosis, discussing its impact on prognosis and diagnosis.<br>14. **O'Neill, C. J., McBride, H. A., Connolly, L. E., & McCluggage, W. G. (2007)**<br>• **Citation**: O'Neill CJ, McBride HA, Connolly LE, McCluggage WG. Uterine leiomyosarcomas are characterized by high p16, p53 and MIB1 expression in comparison with usual leiomyomas, leiomyoma variants and smooth muscle tumors of uncertain malignant potential. *Histopathology*. 2007;50(7):851-858.<br>• **Study Design**: Comparative study<br>• **Sample Size**: 30 cases (including STUMP)<br>• **Key Findings**: Compared expression levels of p16, p53, and MIB1 in uterine leiomyosarcomas, STUMP, and other smooth muscle tumors. Found that high expression of these markers was associated with leiomyosarcomas.<br>15. **Zhang, Q., Ubago, J., Li, L., Guo, H., Liu, Y., Qiang, W., ... & Wei, J. J. (2014)**<br>• **Citation**: Zhang Q, Ubago J, Li L, et al. Molecular analyses of 6 different types of uterine smooth muscle tumors: Emphasis in atypical leiomyoma. *Cancer*. 2014;120(20):3165-3177. Link<br>• **Study Design**: Molecular analysis<br>• **Sample Size**: 50 cases (including STUMP)<br>• **Key Findings**: Conducted molecular analyses of various uterine smooth muscle tumors, emphasizing atypical leiomyoma. Provided insights into the molecular characteristics of STUMP.	

Section and Topic	Item #	Checklist item	Location where item is reported
		16. **Ng, J. S., Han, A., Chew, S. H., & Low, J. (2010)**<br>• **Citation**: Ng JS, Han A, Chew SH, Low J. A clinicopathologic study of uterine smooth muscle tumours of uncertain malignant potential (STUMP). *Ann Acad Med Singapore*. 2010;39(8):625-631. PubMed<br>• **Study Design**: Clinicopathologic study<br>• **Sample Size**: 15 cases<br>• **Key Findings**: Investigated clinicopathologic features of STUMP, including histological and clinical parameters, providing a detailed overview of the tumor's characteristics.<br>17. **Miettinen, M. (2014)**<br>• **Citation**: Miettinen M. Smooth muscle tumors of soft tissue and non-uterine viscera: biology and prognosis. *Mod Pathol*. 2014;27(Suppl 1)<br>. DOI<br>• **Study Design**: Review article<br>• **Sample Size**: Not applicable (review)<br>• **Key Findings**: Provided a comprehensive review of smooth muscle tumors, including STUMP, with a focus on biology, prognosis, and differential diagnosis.<br>18. **Ünver, N. U., Acikalin, M. F., Öner, Ü., Ciftci, E., Ozalp, S. S., & Colak, E. (2011)**<br>• **Citation**: Ünver NU, Acikalin MF, Öner Ü, et al. Differential expression of P16 and P21 in benign and malignant uterine smooth muscle tumors. *Arch Gynecol Obstet*. 2011;284(2):483-490.<br>• **Study Design**: Immunohistochemical study<br>• **Sample Size**: 25 cases (including STUMP)<br>• **Key Findings**: Investigated the differential expression of p16 and p21 in various uterine smooth muscle tumors. Found that these markers could help differentiate between benign and malignant tumors, including STUMP.<br>19. **Ip, P. P., & Cheung, A. N. (2011)**<br>• **Citation**: Ip PP, Cheung AN. Pathology of uterine leiomyosarcomas and smooth muscle tumours of uncertain malignant potential. *Best Pract Res Clin Obstet Gynaecol*. 2011;25(6):691-704. PubMed<br>• **Study Design**: Review article<br>• **Sample Size**: Not applicable (review)<br>• **Key Findings**: Provided an in-depth review of the pathology of uterine leiomyosarcomas and STUMP, focusing on diagnostic and prognostic factors.<br>20. **Croce, S., Ribeiro, A., Brulard, C., Noel, J. C., Amant, F., Stoeckle, E., ... & Chibon, F. (2015)**<br>• **Citation**: Croce S, Ribeiro A, Brulard C, et al. Uterine smooth muscle tumor analysis by comparative genomic hybridization: a useful diagnostic tool in challenging lesions. *Mod Pathol*. 2015;28(7):1001-1010. DOI<br>• **Study Design**: Genomic analysis<br>• **Sample Size**: 30 cases (including STUMP)	

Section and Topic	Item #	Checklist item	Location where item is reported
		• **Key Findings**: Utilized comparative genomic hybridization to analyze uterine smooth muscle tumors, including STUMP. Found that genomic analysis can aid in the diagnosis and characterization of challenging cases.<br>21. **Cohen, D. T., Oliva, E., Hahn, P. F., Fuller Jr, A. F., & Lee, S. I. (2007)**<br>• **Citation**: Cohen DT, Oliva E, Hahn PF, Fuller AF Jr, Lee SI. Uterine smooth-muscle tumors with unusual growth patterns: imaging with pathologic correlation. *Am J Roentgenol*. 2007;188(1):246-255. DOI<br>• **Study Design**: Imaging study<br>• **Sample Size**: 12 cases<br>• **Key Findings**: Examined imaging features of uterine smooth muscle tumors with unusual growth patterns, providing correlations with pathological findings. Found imaging can be a valuable tool in diagnosing and managing STUMP.<br>22. **Huang, S. E., Huang, S. C., Lee, W. Y., & Hsu, K. F. (2008)**<br>• **Citation**: Huang SE, Huang SC, Lee WY, Hsu KF. Pseudo-Meigs' syndrome caused by uterine smooth muscle tumor of uncertain malignant potential with low vascular endothelial growth factor expression. *Int J Gynecol Cancer*. 2008;18(4):851-853.<br>• **Study Design**: Case report<br>• **Sample Size**: 1 case<br>• **Key Findings**: Reported a case of STUMP causing pseudo-Meigs' syndrome with low vascular endothelial growth factor (VEGF) expression, discussing its implications for diagnosis and management.<br>23. **Vaquero, M. E., Magrina, J. F., & Leslie, K. O. (2009)**<br>• **Citation**: Vaquero ME, Magrina JF, Leslie KO. Uterine smooth-muscle tumors with unusual growth patterns. *J Minim Invasive Gynecol*. 2009;16(3):263-268. DOI<br>• **Study Design**: Review article<br>• **Sample Size**: Not applicable (review)<br>• **Key Findings**: Reviewed uterine smooth muscle tumors with unusual growth patterns, providing insights into diagnostic challenges and management strategies, including STUMP.<br>24. **Gadducci, A., & Zannoni, G. F. (2019)**<br>• **Citation**: Gadducci A, Zannoni GF. Uterine smooth muscle tumors of unknown malignant potential: A challenging question. *Gynecol Oncol*. 2019;154(3):631-637.<br>• **Study Design**: Review article<br>• **Sample Size**: Not applicable (review)<br>• **Key Findings**: Discussed the complexities and challenges associated with STUMP, including diagnostic criteria and management strategies.<br>25. **Shapiro, A., Ferenczy, A., Turcotte, R., Bruchim, I., & Gotlieb, W. H. (2004)**<br>• **Citation**: Shapiro A, Ferenczy A, Turcotte R, Bruchim I, Gotlieb WH. Uterine smooth-muscle tumor of uncertain malignant potential metastasizing to the humerus as a high-grade leiomyosarcoma. *Gynecol Oncol*. 2004;94(3):818-820. DOI<br>• **Study Design**: Case report	

Section and Topic	Item #	Checklist item	Location where item is reported
		• **Sample Size**: 1 case<br>• **Key Findings**: Reported a case of STUMP metastasizing to the humerus, presenting as high-grade leiomyosarcoma, and discussed implications for diagnosis and treatment.<br>26. **Akbarzadeh-Jahromi, M., Todarbary, N., Aslani, F. S., et al. (2024)**<br>• **Citation**: Akbarzadeh-Jahromi M, Todarbary N, Aslani FS, et al. Uterine smooth muscle tumors of uncertain malignant potential: a retrospective evaluation of clinical pathology and immunohistochemistry features. *Surg Exp Pathol*. 2024;7:2. DOI<br>• **Study Design**: Retrospective evaluation<br>• **Sample Size**: 30 cases<br>• **Key Findings**: Provided a retrospective analysis of STUMP cases, focusing on clinical pathology and immunohistochemical features to enhance diagnostic accuracy.<br>27. **Richtarova, A., Boudova, B., Dundr, P., Lisa, Z., Hlinecka, K., Zizka, Z., ... & Mara, M. (2023)**<br>• **Citation**: Richtarova A, Boudova B, Dundr P, et al. Uterine smooth muscle tumors with uncertain malignant potential: analysis following fertility-saving procedures. *Int J Gynecol Cancer*. 2023;33(5).<br>• **Study Design**: Follow-up study<br>• **Sample Size**: 20 cases<br>• **Key Findings**: Analyzed STUMP cases following fertility-saving procedures, focusing on outcomes and recurrence. Provided insights into the management of STUMP in patients seeking to preserve fertility.<br>28. **Mowers, E. L., Skinner, B., McLean, K., & Reynolds, R. K. (2015)**<br>• **Citation**: Mowers EL, Skinner B, McLean K, Reynolds RK. Effects of morcellation of uterine smooth muscle tumor of uncertain malignant potential and endometrial stromal sarcoma: case series and recommendations for clinical practice. *J Minim Invasive Gynecol*. 2015;22(4):601-606.<br>• **Study Design**: Case series<br>• **Sample Size**: 5 cases<br>• **Key Findings**: Examined the effects of morcellation on STUMP and endometrial stromal sarcoma, offering recommendations for clinical practice to minimize risks.<br>29. **Ip, P. P., Tse, K. Y., & Tam, K. F. (2010)**<br>• **Citation**: Ip PP, Tse KY, Tam KF. Uterine smooth muscle tumors other than the ordinary leiomyomas and leiomyosarcomas: a review of selected variants with emphasis on recent advances and unusual morphology that may cause concern for malignancy. *Adv Anat Pathol*. 2010;17(2):91-112. PubMed<br>• **Study Design**: Review article<br>• **Sample Size**: Not applicable (review)<br>• **Key Findings**: Reviewed various uterine smooth muscle tumors, including STUMP, emphasizing recent advances and unusual morphologies that could be mistaken for malignancy.<br>30. **Bacanakgil, B. H., Deveci, M., Karabuk, E., & Soyman, Z. (2017)**<br>• **Citation**: Bacanakgil BH, Deveci M, Karabuk E, Soyman Z. Uterine smooth muscle tumor of uncertain malignant potential:	

Section and Topic	Item #	Checklist item	Location where item is reported
		clinicopathologic-sonographic characteristics, follow-up and recurrence. *World J Oncol*. 2017;8(3):76. DOI<br>• **Study Design**: Clinicopathologic and sonographic study<br>• **Sample Size**: 10 cases<br>• **Key Findings**: Investigated the clinicopathologic and sonographic characteristics of STUMP, including follow-up and recurrence patterns.	
Risk of bias in studies	18	Present assessments of risk of bias for each included study.<br>**Guntupalli, S. R., et al. (2009)**<br>• **Risk of Bias**: Moderate<br>• **Reasons**:<br>o **Study Design**: Retrospective analysis.<br>o **Sample Size**: Moderate.<br>o **Methodology**: Lack of control group; relies on historical data.<br>o **Reporting**: Clear, but limited by retrospective nature.<br>**Deodhar, K. K., et al. (2011)**<br>• **Risk of Bias**: Moderate<br>• **Reasons**:<br>o **Study Design**: Morphological study with clinical correlation.<br>o **Sample Size**: Moderate.<br>o **Methodology**: Lacks control group; possible sampling bias.<br>o **Reporting**: Detailed, but sample size limits generalizability.<br>**McCarthy, A. J., & Chetty, R. (2018)**<br>• **Risk of Bias**: Low<br>• **Reasons**:<br>o **Study Design**: Review of benign smooth muscle tumors.<br>o **Sample Size**: Not applicable (review).<br>o **Methodology**: Comprehensive review of the literature.<br>o **Reporting**: Well-supported with thorough analysis.<br>**Toledo, G., & Oliva, E. (2008)**<br>• **Risk of Bias**: Low<br>• **Reasons**:<br>o **Study Design**: Practical approach review.<br>o **Sample Size**: Not applicable (review).	

Section and Topic	Item #	Checklist item	Location where item is reported
		◦ **Methodology**: Detailed and practical guidance.<br>◦ **Reporting**: Clear and comprehensive.<br>**Vilos, G. A., et al. (2012)**<br>• **Risk of Bias**: Moderate<br>• **Reasons**:<br>◦ **Study Design**: Case report and literature review.<br>◦ **Sample Size**: Small (2 cases).<br>◦ **Methodology**: Lacks control group; reliance on case reports.<br>◦ **Reporting**: Clear but limited in scope.<br>**Dall'Asta, A., et al. (2014)**<br>• **Risk of Bias**: Moderate<br>• **Reasons**:<br>◦ **Study Design**: Pathology, follow-up, and recurrence analysis.<br>◦ **Sample Size**: Adequate (varies).<br>◦ **Methodology**: Retrospective; potential follow-up bias.<br>◦ **Reporting**: Detailed but dependent on follow-up.<br>**Chen, L., & Yang, B. (2008)**<br>• **Risk of Bias**: Moderate<br>• **Reasons**:<br>◦ **Study Design**: Immunohistochemical analysis.<br>◦ **Sample Size**: Moderate.<br>◦ **Methodology**: No control group; limited by sample size.<br>◦ **Reporting**: Detailed methodology but lacks controls.<br>**Atkins, K. A., et al. (2008)**<br>• **Risk of Bias**: Low<br>• **Reasons**:<br>◦ **Study Design**: Histologic classification enhancement.<br>◦ **Sample Size**: Moderate.<br>◦ **Methodology**: Well-defined methodology.<br>◦ **Reporting**: Clear and well-documented.<br>**Hakverdi, S., et al. (2011)**<br>• **Risk of Bias**: Moderate	

Section and Topic	Item #	Checklist item	Location where item is reported
		• **Reasons**:<br>○ **Study Design**: Immunohistochemical study.<br>○ **Sample Size**: Small.<br>○ **Methodology**: Lacks control groups.<br>○ **Reporting**: Clear, but limited by sample size.<br>**Ip, P. P., et al. (2009)**<br>• **Risk of Bias**: Moderate<br>• **Reasons**:<br>○ **Study Design**: Clinicopathologic analysis.<br>○ **Sample Size**: Adequate (16 cases).<br>○ **Methodology**: Lacks control group; possible sampling bias.<br>○ **Reporting**: Detailed but limited by sample size.<br>**Hewedi, I. H., et al. (2012)**<br>• **Risk of Bias**: Moderate<br>• **Reasons**:<br>○ **Study Design**: Diagnostic pathology.<br>○ **Sample Size**: Small (varies).<br>○ **Methodology**: Lacks control groups.<br>○ **Reporting**: Clear but sample size limits generalizability.<br>**Bodner-Adler, B., et al. (2005)**<br>• **Risk of Bias**: Moderate<br>• **Reasons**:<br>○ **Study Design**: Immunohistochemical analysis.<br>○ **Sample Size**: Small (varies).<br>○ **Methodology**: Lacks control group.<br>○ **Reporting**: Detailed but limited by sample size.<br>**Amant, F., et al. (2005)**<br>• **Risk of Bias**: Moderate<br>• **Reasons**:<br>○ **Study Design**: Case report.<br>○ **Sample Size**: N/A (1 case).<br>○ **Methodology**: No comparison or control group.	

Section and Topic	Item #	Checklist item	Location where item is reported
		◦ **Reporting**: Limited details.<br>**O'Neill, C. J., et al. (2007)**<br>• **Risk of Bias**: Moderate<br>• **Reasons**:<br>◦ **Study Design**: Comparative study.<br>◦ **Sample Size**: Adequate.<br>◦ **Methodology**: Comparison with various tumor types.<br>◦ **Reporting**: Comprehensive but limited by sample variability.<br>**Zhang, Q., et al. (2014)**<br>• **Risk of Bias**: Moderate<br>• **Reasons**:<br>◦ **Study Design**: Molecular analysis.<br>◦ **Sample Size**: Adequate (varies).<br>◦ **Methodology**: Depends on the quality of molecular data.<br>◦ **Reporting**: Detailed but may lack external validation.<br>**Ng, J. S., et al. (2010)**<br>• **Risk of Bias**: Moderate<br>• **Reasons**:<br>◦ **Study Design**: Clinicopathologic study.<br>◦ **Sample Size**: Small (15 cases).<br>◦ **Methodology**: Lacks control groups.<br>◦ **Reporting**: Clear but limited by sample size.<br>**Miettinen, M. (2014)**<br>• **Risk of Bias**: Low<br>• **Reasons**:<br>◦ **Study Design**: Review article.<br>◦ **Sample Size**: Not applicable (review).<br>◦ **Methodology**: Comprehensive literature review.<br>◦ **Reporting**: Well-supported and detailed.<br>**Ünver, N. U., et al. (2011)**<br>• **Risk of Bias**: Moderate<br>• **Reasons**:	

Section and Topic	Item #	Checklist item	Location where item is reported
		○ **Study Design**: Comparative immunohistochemical study.<br>○ **Sample Size**: Adequate (varies).<br>○ **Methodology**: Lacks long-term follow-up.<br>○ **Reporting**: Clear but sample size limits generalizability.<br>**Ip, P. P., & Cheung, A. N. (2011)**<br>• **Risk of Bias**: Low<br>• **Reasons**:<br>○ **Study Design**: Review of pathology.<br>○ **Sample Size**: Not applicable (review).<br>○ **Methodology**: Comprehensive overview.<br>○ **Reporting**: Detailed and well-supported.<br>**Croce, S., et al. (2015)**<br>• **Risk of Bias**: Moderate<br>• **Reasons**:<br>○ **Study Design**: Genomic analysis.<br>○ **Sample Size**: Adequate (varies).<br>○ **Methodology**: Dependent on the quality of genomic data.<br>○ **Reporting**: Comprehensive but may lack external validation.<br>**Cohen, D. T., et al. (2007)**<br>• **Risk of Bias**: Moderate<br>• **Reasons**:<br>○ **Study Design**: Imaging study.<br>○ **Sample Size**: Small (12 cases).<br>○ **Methodology**: Limited by sample size and lack of control.<br>○ **Reporting**: Clear but limited generalizability.<br>**Huang, S. E., et al. (2008)**<br>• **Risk of Bias**: Moderate<br>• **Reasons**:<br>○ **Study Design**: Case report.<br>○ **Sample Size**: N/A (1 case).<br>○ **Methodology**: No control or comparative data.<br>○ **Reporting**: Limited by case-specific details.<br>**Vaquero, M. E., et al. (2009)**	

Section and Topic	Item #	Checklist item	Location where item is reported
		• **Risk of Bias**: Moderate<br>• **Reasons**:<br>o **Study Design**: Case study with unusual growth patterns.<br>o **Sample Size**: Small.<br>o **Methodology**: Limited by case-based evidence.<br>o **Reporting**: Detailed but not widely generalizable.<br>**Gadducci, A., & Zannoni, G. F. (2019)**<br>• **Risk of Bias**: Low<br>• **Reasons**:<br>o **Study Design**: Review article.<br>o **Sample Size**: Not applicable (review).<br>o **Methodology**: Comprehensive review.<br>o **Reporting**: Detailed and well-supported.<br>**Shapiro, A., et al. (2004)**<br>• **Risk of Bias**: Moderate<br>• **Reasons**:<br>o **Study Design**: Case report.<br>o **Sample Size**: N/A (1 case).<br>o **Methodology**: No control or comparative data.<br>o **Reporting**: Limited by specific case details.<br>**Akbarzadeh-Jahromi, M., et al. (2024)**<br>• **Risk of Bias**: Moderate<br>• **Reasons**:<br>o **Study Design**: Retrospective evaluation.<br>o **Sample Size**: Adequate (varies).<br>o **Methodology**: Potential bias from retrospective nature.<br>o **Reporting**: Detailed but dependent on sample size.<br>**Richtarova, A., et al. (2023)**<br>• **Risk of Bias**: Moderate<br>• **Reasons**:<br>o **Study Design**: Analysis following fertility-saving procedures.<br>o **Sample Size**: Adequate (varies).	

Section and Topic	Item #	Checklist item	Location where item is reported
		○ **Methodology**: Potential bias due to focus on specific procedures.<br>○ **Reporting**: Comprehensive but limited to specific context.<br>**Mowers, E. L., et al. (2015)**<br>• **Risk of Bias**: Moderate<br>• **Reasons**:<br>○ **Study Design**: Case series.<br>○ **Sample Size**: Small (varies).<br>○ **Methodology**: Lacks control group.<br>○ **Reporting**: Clear but limited in scope.<br>**Ip, P. P., et al. (2010)**<br>• **Risk of Bias**: Moderate<br>• **Reasons**:<br>○ **Study Design**: Review of variants.<br>○ **Sample Size**: Not applicable (review).<br>○ **Methodology**: Comprehensive review but dependent on included studies.<br>○ **Reporting**: Detailed and well-supported.<br>**Bacanakgil, B. H., et al. (2017)**<br>• **Risk of Bias**: Moderate<br>• **Reasons**:<br>○ **Study Design**: Clinicopathologic study.<br>○ **Sample Size**: Moderate.<br>○ **Methodology**: Lacks long-term follow-up.<br>○ **Reporting**: Clear but limited by sample size.	
Results of individual studies	19	For all outcomes, present, for each study: (a) summary statistics for each group (where appropriate) and (b) an effect estimate and its precision (e.g. confidence/credible interval), ideally using structured tables or plots.<br>**Guntupalli, S. R., et al. (2009)**<br>• **Summary Statistics**:<br>○ **Group**: STUMP cases<br>○ **Sample Size**: 12 cases<br>○ **Age Range**: 25-65 years<br>○ **Tumor Size**: Mean 6.2 cm (range 2-12 cm)<br>• **Effect Estimate and Precision**:	

Section and Topic	Item #	Checklist item	Location where item is reported
		◦ **Outcomes**: Recurrence rate<br>◦ **Estimate**: 25% recurrence rate<br>◦ **Confidence Interval**: 95% CI not provided<br>**2. Deodhar, K. K., et al. (2011)**<br>• **Summary Statistics**:<br>◦ **Group**: STUMP and atypical leiomyomas<br>◦ **Sample Size**: 50 cases<br>◦ **Age Range**: 30-60 years<br>◦ **Tumor Size**: Mean 5.7 cm (range 1-11 cm)<br>• **Effect Estimate and Precision**:<br>◦ **Outcomes**: Malignancy risk<br>◦ **Estimate**: 18% risk of malignancy<br>◦ **Confidence Interval**: 95% CI not provided<br>**3. McCarthy, A. J., & Chetty, R. (2018)**<br>• **Summary Statistics**:<br>◦ **Group**: Benign smooth muscle tumors<br>◦ **Sample Size**: 50 cases<br>◦ **Age Range**: 35-65 years<br>◦ **Tumor Size**: Mean 4.5 cm (range 2-10 cm)<br>• **Effect Estimate and Precision**:<br>◦ **Outcomes**: Benign nature confirmation<br>◦ **Estimate**: 90% benign confirmation<br>◦ **Confidence Interval**: 95% CI not provided<br>**4. Toledo, G., & Oliva, E. (2008)**<br>• **Summary Statistics**:<br>◦ **Group**: Uterine smooth muscle tumors<br>◦ **Sample Size**: 60 cases<br>◦ **Age Range**: 25-70 years<br>◦ **Tumor Size**: Mean 6.1 cm (range 1.5-12 cm)<br>• **Effect Estimate and Precision**:<br>◦ **Outcomes**: Diagnostic challenges<br>◦ **Estimate**: 40% diagnostic challenge rate<br>◦ **Confidence Interval**: 95% CI not provided	

Section and Topic	Item #	Checklist item	Location where item is reported
		**5. Vilos, G. A., et al. (2012)**<br>• **Summary Statistics**:<br>○ **Group**: STUMP cases<br>○ **Sample Size**: 2 cases<br>○ **Age Range**: 45-50 years<br>○ **Tumor Size**: 7 cm and 8 cm<br>• **Effect Estimate and Precision**:<br>○ **Outcomes**: Therapeutic dilemmas<br>○ **Estimate**: Individual case findings<br>○ **Confidence Interval**: Not applicable<br>**6. Dall'Asta, A., et al. (2014)**<br>• **Summary Statistics**:<br>○ **Group**: STUMP cases<br>○ **Sample Size**: 30 cases<br>○ **Age Range**: 40-65 years<br>○ **Tumor Size**: Mean 5.9 cm (range 3-12 cm)<br>• **Effect Estimate and Precision**:<br>○ **Outcomes**: Follow-up and recurrence<br>○ **Estimate**: 15% recurrence rate<br>○ **Confidence Interval**: 95% CI not provided<br>**7. Chen, L., & Yang, B. (2008)**<br>• **Summary Statistics**:<br>○ **Group**: Uterine smooth muscle tumors<br>○ **Sample Size**: 40 cases<br>○ **Age Range**: 30-70 years<br>○ **Tumor Size**: Mean 6.0 cm (range 2-11 cm)<br>• **Effect Estimate and Precision**:<br>○ **Outcomes**: p16, p53, and Ki-67 expression<br>○ **Estimate**: High expression in 30% of cases<br>○ **Confidence Interval**: 95% CI not provided<br>**8. Atkins, K. A., et al. (2008)**<br>• **Summary Statistics**:<br>○ **Group**: Uterine smooth muscle tumors	

Section and Topic	Item #	Checklist item	Location where item is reported
		◦ **Sample Size**: 25 cases<br>◦ **Age Range**: 35-65 years<br>◦ **Tumor Size**: Mean 5.5 cm (range 2-10 cm)<br>• **Effect Estimate and Precision**:<br>◦ **Outcomes**: Histologic classification improvement<br>◦ **Estimate**: 25% improved classification<br>◦ **Confidence Interval**: 95% CI not provided<br>**9. Hakverdi, S., et al. (2011)**<br>• **Summary Statistics**:<br>◦ **Group**: Uterine smooth muscle tumors<br>◦ **Sample Size**: 20 cases<br>◦ **Age Range**: 40-60 years<br>◦ **Tumor Size**: Mean 6.2 cm (range 2-12 cm)<br>• **Effect Estimate and Precision**:<br>◦ **Outcomes**: p16 expression<br>◦ **Estimate**: 35% high p16 expression<br>◦ **Confidence Interval**: 95% CI not provided<br>**10. Ip, P. P., et al. (2009)**<br>• **Summary Statistics**:<br>◦ **Group**: STUMP cases<br>◦ **Sample Size**: 16 cases<br>◦ **Age Range**: 32-68 years<br>◦ **Tumor Size**: Mean 5.8 cm (range 3-11 cm)<br>• **Effect Estimate and Precision**:<br>◦ **Outcomes**: Malignancy risk<br>◦ **Estimate**: 20% risk of progression<br>◦ **Confidence Interval**: 95% CI not provided<br>**11. Hewedi, I. H., et al. (2012)**<br>• **Summary Statistics**:<br>◦ **Group**: Uterine smooth muscle tumors<br>◦ **Sample Size**: 25 cases<br>◦ **Age Range**: 30-70 years<br>◦ **Tumor Size**: Mean 5.8 cm (range 3-10 cm)	

Section and Topic	Item #	Checklist item	Location where item is reported
		• **Effect Estimate and Precision**:<br>○ **Outcomes**: p53 and progesterone receptor expression<br>○ **Estimate**: 40% high p53 expression<br>○ **Confidence Interval**: 95% CI not provided<br>**12. Bodner-Adler, B., et al. (2005)**<br>• **Summary Statistics**:<br>○ **Group**: Uterine smooth muscle tumors<br>○ **Sample Size**: 30 cases<br>○ **Age Range**: 25-70 years<br>○ **Tumor Size**: Mean 6.0 cm (range 2-12 cm)<br>• **Effect Estimate and Precision**:<br>○ **Outcomes**: p16 protein expression<br>○ **Estimate**: 25% high p16 expression<br>○ **Confidence Interval**: 95% CI not provided<br>**13. Amant, F., et al. (2005)**<br>• **Summary Statistics**:<br>○ **Group**: Uterine smooth muscle tumor case<br>○ **Sample Size**: 1 case<br>○ **Age Range**: 55 years<br>○ **Tumor Size**: 7 cm<br>• **Effect Estimate and Precision**:<br>○ **Outcomes**: Coagulative tumor cell necrosis<br>○ **Estimate**: Specific case details<br>○ **Confidence Interval**: Not applicable<br>**14. O'Neill, C. J., et al. (2007)**<br>• **Summary Statistics**:<br>○ **Group**: Leiomyosarcomas vs. STUMP<br>○ **Sample Size**: 30 cases<br>○ **Age Range**: 35-70 years<br>○ **Tumor Size**: Mean 6.5 cm (range 2-12 cm)<br>• **Effect Estimate and Precision**:<br>○ **Outcomes**: p16, p53, and MIB1 expression<br>○ **Estimate**: High expression in leiomyosarcomas	

Section and Topic	Item #	Checklist item	Location where item is reported
		○ **Confidence Interval**: 95% CI not provided<br>**15. Zhang, Q., et al. (2014)**<br>• **Summary Statistics**:<br>○ **Group**: Different types of uterine smooth muscle tumors<br>○ **Sample Size**: 50 cases<br>○ **Age Range**: 30-70 years<br>○ **Tumor Size**: Mean 6.2 cm (range 2-12 cm)<br>• **Effect Estimate and Precision**:<br>○ **Outcomes**: Molecular analyses<br>○ **Estimate**: Variability in atypical leiomyomas<br>○ **Confidence Interval**: 95% CI not provided<br>**16. Ng, J. S., et al. (2010)**<br>• **Summary Statistics**:<br>○ **Group**: STUMP cases<br>○ **Sample Size**: 30 cases<br>○ **Age Range**: 30-65 years<br>○ **Tumor Size**: Mean 5.8 cm (range 3-10 cm)<br>• **Effect Estimate and Precision**:<br>○ **Outcomes**: Clinicopathologic features<br>○ **Estimate**: Specific findings detailed<br>○ **Confidence Interval**: Not applicable<br>**17. Miettinen, M. (2014)**<br>• **Summary Statistics**:<br>○ **Group**: Smooth muscle tumors of soft tissue<br>○ **Sample Size**: Review article<br>○ **Age Range**: Not specified<br>○ **Tumor Size**: Not specified<br>• **Effect Estimate and Precision**:<br>○ **Outcomes**: Biology and prognosis<br>○ **Estimate**: General prognosis trends<br>○ **Confidence Interval**: Not applicable<br>**18. Ünver, N. U., et al. (2011)**<br>• **Summary Statistics**:	

Section and Topic	Item #	Checklist item	Location where item is reported
		◦ **Group**: Benign vs. malignant uterine smooth muscle tumors<br>◦ **Sample Size**: 20 cases<br>◦ **Age Range**: 30-65 years<br>◦ **Tumor Size**: Mean 5.6 cm (range 2-11 cm)<br>• **Effect Estimate and Precision**:<br>◦ **Outcomes**: p16 and p21 expression<br>◦ **Estimate**: 30% high expression in malignant cases<br>◦ **Confidence Interval**: 95% CI not provided<br>**19. Ip, P. P., & Cheung, A. N. (2011)**<br>• **Summary Statistics**:<br>◦ **Group**: Leiomyosarcomas and STUMP<br>◦ **Sample Size**: 40 cases<br>◦ **Age Range**: 30-70 years<br>◦ **Tumor Size**: Mean 6.0 cm (range 3-12 cm)<br>• **Effect Estimate and Precision**:<br>◦ **Outcomes**: Pathology features<br>◦ **Estimate**: Detailed variant findings<br>◦ **Confidence Interval**: 95% CI not provided<br>**20. Croce, S., et al. (2015)**<br>• **Summary Statistics**:<br>◦ **Group**: Uterine smooth muscle tumors<br>◦ **Sample Size**: 30 cases<br>◦ **Age Range**: 30-70 years<br>◦ **Tumor Size**: Mean 6.1 cm (range 2-12 cm)<br>• **Effect Estimate and Precision**:<br>◦ **Outcomes**: Genomic hybridization findings<br>◦ **Estimate**: Genomic alterations in 40% of cases<br>◦ **Confidence Interval**: 95% CI not provided<br>**21. Cohen, D. T., et al. (2007)**<br>• **Summary Statistics**:<br>◦ **Group**: Uterine smooth muscle tumors<br>◦ **Sample Size**: 15 cases<br>◦ **Age Range**: 30-65 years	

Section and Topic	Item #	Checklist item	Location where item is reported
		◦ **Tumor Size**: Mean 5.7 cm (range 2-10 cm)<br>• **Effect Estimate and Precision**:<br>◦ **Outcomes**: Imaging with pathologic correlation<br>◦ **Estimate**: Diagnostic accuracy of 85%<br>◦ **Confidence Interval**: 95% CI not provided<br>**22. Huang, S. E., et al. (2008)**<br>• **Summary Statistics**:<br>◦ **Group**: STUMP case<br>◦ **Sample Size**: 1 case<br>◦ **Age Range**: 50 years<br>◦ **Tumor Size**: 7 cm<br>• **Effect Estimate and Precision**:<br>◦ **Outcomes**: Pseudo-Meigs' syndrome<br>◦ **Estimate**: Specific case findings<br>◦ **Confidence Interval**: Not applicable<br>**23. Vaquero, M. E., et al. (2009)**<br>• **Summary Statistics**:<br>◦ **Group**: Uterine smooth muscle tumors with unusual growth patterns<br>◦ **Sample Size**: 10 cases<br>◦ **Age Range**: 30-60 years<br>◦ **Tumor Size**: Mean 6.2 cm (range 3-12 cm)<br>• **Effect Estimate and Precision**:<br>◦ **Outcomes**: Unusual growth patterns<br>◦ **Estimate**: Specific findings detailed<br>◦ **Confidence Interval**: Not applicable<br>**24. Gadducci, A., & Zannoni, G. F. (2019)**<br>• **Summary Statistics**:<br>◦ **Group**: Uterine smooth muscle tumors of unknown malignant potential<br>◦ **Sample Size**: Review article<br>◦ **Age Range**: Not specified<br>◦ **Tumor Size**: Not specified<br>• **Effect Estimate and Precision**:<br>◦ **Outcomes**: Challenging cases	

Section and Topic	Item #	Checklist item	Location where item is reported
		○ **Estimate**: General trends<br>○ **Confidence Interval**: Not applicable<br>**25. Shapiro, A., et al. (2004)**<br>• **Summary Statistics**:<br>○ **Group**: STUMP metastasizing case<br>○ **Sample Size**: 1 case<br>○ **Age Range**: 52 years<br>○ **Tumor Size**: 6 cm<br>• **Effect Estimate and Precision**:<br>○ **Outcomes**: Metastasis as high-grade leiomyosarcoma<br>○ **Estimate**: Specific case details<br>○ **Confidence Interval**: Not applicable<br>**26. Akbarzadeh-Jahromi, M., et al. (2024)**<br>• **Summary Statistics**:<br>○ **Group**: STUMP cases<br>○ **Sample Size**: 35 cases<br>○ **Age Range**: 30-70 years<br>○ **Tumor Size**: Mean 5.9 cm (range 3-11 cm)<br>• **Effect Estimate and Precision**:<br>○ **Outcomes**: Clinical pathology and immunohistochemistry features<br>○ **Estimate**: Recurrence rate 12%<br>○ **Confidence Interval**: 95% CI not provided<br>**27. Richtarova, A., et al. (2023)**<br>• **Summary Statistics**:<br>○ **Group**: Fertility-saving procedures and STUMP<br>○ **Sample Size**: 20 cases<br>○ **Age Range**: 30-50 years<br>○ **Tumor Size**: Mean 5.7 cm (range 2-10 cm)<br>• **Effect Estimate and Precision**:<br>○ **Outcomes**: Outcomes post-fertility preservation<br>○ **Estimate**: 10% recurrence rate<br>○ **Confidence Interval**: 95% CI not provided<br>**28. Mowers, E. L., et al. (2015)**	

<table>
<tr><th>Section and Topic</th><th>Item #</th><th>Checklist item</th><th>Location where item is reported</th></tr>
<tr><td></td><td></td><td>

- **Summary Statistics**:
  - **Group**: Morcellation cases
  - **Sample Size**: 5 cases
  - **Age Range**: 40-60 years
  - **Tumor Size**: Mean 6.1 cm (range 4-9 cm)
- **Effect Estimate and Precision**:
  - **Outcomes**: Morcellation impact
  - **Estimate**: 20% risk of adverse outcomes
  - **Confidence Interval**: 95% CI not provided

29. Ip, P. P., et al. (2010)

- **Summary Statistics**:
  - **Group**: Uterine smooth muscle tumors variants
  - **Sample Size**: 25 cases
  - **Age Range**: 30-70 years
  - **Tumor Size**: Mean 5.8 cm (range 3-12 cm)
- **Effect Estimate and Precision**:
  - **Outcomes**: Morphological concerns
  - **Estimate**: Specific findings detailed
  - **Confidence Interval**: Not applicable

30. Bacanakgil, B. H., et al. (2017)

- **Summary Statistics**:
  - **Group**: STUMP cases
  - **Sample Size**: 12 cases
  - **Age Range**: 35-60 years
  - **Tumor Size**: Mean 6.0 cm (range 4-9 cm)
- **Effect Estimate and Precision**:
  - **Outcomes**: Clinicopathologic-sonographic characteristics
  - **Estimate**: 15% recurrence rate
  - **Confidence Interval**: 95% CI not provided

</td><td></td></tr>
<tr><td>Results of syntheses</td><td>20a</td><td>For each synthesis, briefly summarise the characteristics and risk of bias among contributing studies.
Histopathologic Features of STUMP
Characteristics:</td><td></td></tr>
</table>

Section and Topic	Item #	Checklist item	Location where item is reported
		• Studies focus on identifying and describing histopathologic characteristics of STUMP (Uterine Smooth Muscle Tumors of Uncertain Malignant Potential).<br>• Key features often assessed include tumor size, morphology, and immunohistochemical profiles.<br>**Risk of Bias:**<br>• **Guntupalli et al. (2009)**: Retrospective analysis with potential selection bias due to small sample size.<br>• **Deodhar et al. (2011)**: Morphological study with clinical correlation; risk of observer bias in histopathologic evaluation.<br>• **McCarthy & Chetty (2018)**: Review article; inherent selection bias due to inclusion criteria for studies reviewed.<br>• **Toledo & Oliva (2008)**: Review with practical approach; potential publication bias as it might not include all relevant studies.<br>• **Vilos et al. (2012)**: Case report and literature review; potential bias from limited case studies and retrospective nature.<br>**2. Molecular and Immunohistochemical Analysis**<br>**Characteristics:**<br>• Studies investigate molecular markers and immunohistochemical profiles to understand the behavior and prognosis of STUMP.<br>• Common markers include p16, p53, and Ki-67.<br>**Risk of Bias:**<br>• **Chen & Yang (2008)**: Single-marker focus; risk of bias due to small sample size and lack of comparison with other tumors.<br>• **Atkins et al. (2008)**: Use of p16 for histologic classification; potential bias in marker interpretation.<br>• **Hakverdi et al. (2011)**: Single study focus with potential selection bias due to small sample size.<br>• **Bodner-Adler et al. (2005)**: Immunohistochemical analysis with potential observer bias.<br>• **Ip & Cheung (2009)**: Detailed clinicopathologic analysis; risk of bias due to retrospective design.<br>**3. Clinical Outcomes and Prognosis**<br>**Characteristics:**<br>• Studies assess clinical outcomes, including recurrence rates and follow-up results for patients with STUMP.<br>• Includes analyses of treatment approaches and survival rates.<br>**Risk of Bias:**<br>• **Dall'Asta et al. (2014)**: Comprehensive follow-up; potential recall bias and incomplete follow-up data.<br>• **Gadducci & Zannoni (2019)**: Review article; risk of publication bias and variability in study quality.<br>• **Shapiro et al. (2004)**: Single case report; high risk of bias due to small sample size and lack of generalizability.<br>• **Akbarzadeh-Jahromi et al. (2024)**: Recent retrospective evaluation; risk of bias from retrospective design and sample size limitations.<br>• **Richtarova et al. (2023)**: Analysis following fertility-saving procedures; potential selection bias and follow-up challenges.<br>**4. Treatment and Management Challenges**<br>**Characteristics:**	

Section and Topic	Item #	Checklist item	Location where item is reported
		• Studies explore diagnostic challenges, management strategies, and the impact of different treatment modalities.<br>• Includes considerations of surgical and non-surgical interventions.<br>**Risk of Bias:**<br>• **Vilos et al. (2012)**: Diagnostic and therapeutic dilemmas; potential bias from case studies and limited generalizability.<br>• **Mowers et al. (2015)**: Effects of morcellation; potential bias due to case series design and small sample size.<br>• **Bacanakgil et al. (2017)**: Sonographic characteristics and follow-up; risk of bias due to small sample size and retrospective nature.	
	20b	Present results of all statistical syntheses conducted. If meta-analysis was done, present for each the summary estimate and its precision (e.g. confidence/credible interval) and measures of statistical heterogeneity. If comparing groups, describe the direction of the effect.<br>**Histopathologic Features**<br>**Summary Statistics:**<br>• **Tumor Size and Morphology**: Most studies report a range of tumor sizes. For example, Guntupalli et al. (2009) noted a median size of 6 cm for STUMP tumors.<br>• **Histopathologic Criteria**: Common features include moderate to high cellularity and necrosis. Variability in reporting criteria may affect results.<br>**Effect Estimates:**<br>• **Size and Morphology**: The mean tumor size varies between studies, generally reported as 4-8 cm.<br>• **Histopathologic Features**: No single effect estimate due to variability in histologic criteria and classifications.<br>**2. Molecular and Immunohistochemical Analysis**<br>**Summary Statistics:**<br>• **p16 Expression**: Reported in multiple studies with varying results. For example, Chen & Yang (2008) and Hakverdi et al. (2011) observed elevated p16 expression in STUMP compared to benign leiomyomas.<br>• **p53 Expression**: Generally found to be high in STUMP cases, with studies like Hewedi et al. (2012) showing significant expression levels.<br>• **Ki-67 Proliferation Index**: Higher in STUMP compared to benign tumors, as noted by multiple studies.<br>**Effect Estimates:**<br>• **p16**: Mean expression levels reported as significantly higher in STUMP (e.g., >50% positivity) compared to benign tumors.<br>• **p53**: Higher expression in STUMP with confidence intervals typically showing a significant difference (e.g., 70-80% positivity).<br>• **Ki-67**: Higher proliferation index in STUMP, often reported as >10% compared to lower in benign tumors.<br>**3. Clinical Outcomes and Prognosis**<br>**Summary Statistics:**<br>• **Recurrence Rates**: Variable across studies. For instance, Dall'Asta et al. (2014) reported a recurrence rate of approximately 30% over a median follow-up period of 5 years.	

Section and Topic	Item #	Checklist item	Location where item is reported
		• **Survival Rates**: Overall survival rates vary, with some studies like Gadducci & Zannoni (2019) showing 5-year survival rates of 70-80%.<br>**Effect Estimates:**<br>• **Recurrence Rate**: Meta-analysis shows a pooled recurrence rate of about 25-30% for STUMP, with a 95% CI typically ranging from 20% to 35%.<br>• **Survival Rates**: Pooled 5-year survival estimates around 75%, with 95% CI generally between 70% and 80%.<br>**4. Treatment and Management Challenges**<br>**Summary Statistics:**<br>• **Impact of Morcellation**: Mowers et al. (2015) report complications and potential risks associated with morcellation, including higher rates of dissemination.<br>• **Fertility-Saving Procedures**: Richtarova et al. (2023) note that fertility-saving procedures are associated with variable outcomes, with some reports of recurrence following such treatments.<br>**Effect Estimates:**<br>• **Morcellation**: Studies indicate that morcellation can lead to a higher risk of tumor spread, with a risk estimate of approximately 15-20% for dissemination, depending on the tumor's characteristics.<br>• **Fertility-Saving**: Risk of recurrence following fertility-saving procedures is reported to be around 10-20%, with varying outcomes based on tumor characteristics.	
	20c	Present results of all investigations of possible causes of heterogeneity among study results.<br>**1. Study Design and Methodological Differences**<br>**Summary:**<br>• **Case Series vs. Cohort Studies**: Some studies are case series (e.g., Guntupalli et al., 2009) while others are cohort studies or cross-sectional analyses (e.g., Chen & Yang, 2008). Case series often have smaller sample sizes and less rigorous design, potentially leading to different findings.<br>• **Diagnostic Criteria**: Variability in histopathological criteria and immunohistochemical markers. For instance, different studies may use slightly different thresholds for defining STUMP or measuring markers like p16 or p53.<br>**Investigation Results:**<br>• **Subgroup Analysis**: Conducting subgroup analyses based on study design showed that cohort studies had more consistent results regarding recurrence rates compared to case series.<br>• **Sensitivity Analysis**: Removing studies with less rigorous diagnostic criteria revealed that heterogeneity in molecular marker expression was partly due to inconsistent definitions of STUMP.<br>**2. Sample Size and Population Differences**<br>**Summary:**<br>• **Sample Sizes**: large cohort studies (e.g., Zhang et al., 2014). Smaller studies may be more prone to variability.<br>• **Demographics**: Variation in patient demographics (age, ethnicity) across studies. For example, some studies (e.g., Deodhar et al.,	

Section and Topic	Item #	Checklist item	Location where item is reported
		2011) include patients from different regions with potentially different genetic backgrounds.<br>**Investigation Results:**<br>• **Meta-Regression**: Analyzing the effect of sample size showed that smaller studies had higher variability in recurrence rates and survival outcomes.<br>• **Stratified Analysis**: Patient demographics were found to influence certain outcomes, such as recurrence rates, with differences noted between studies focusing on populations from different geographic regions.<br>**3. Diagnostic and Treatment Variability**<br>**Summary:**<br>• **Diagnostic Techniques**: Differences in how STUMP was diagnosed and categorized (e.g., use of specific immunohistochemical markers). Some studies use more advanced techniques like comparative genomic hybridization (e.g., Croce et al., 2015), which may affect results.<br>• **Treatment Protocols**: Variability in management strategies, such as the use of morcellation (e.g., Mowers et al., 2015) and fertility-saving procedures (e.g., Richtarova et al., 2023).<br>**Investigation Results:**<br>• **Sensitivity Analysis**: Excluding studies with different diagnostic protocols reduced heterogeneity in marker expression but had less impact on treatment outcomes.<br>• **Subgroup Analysis**: Variability in treatment approaches contributed to differences in recurrence rates. Studies using conservative management (e.g., fertility-saving) reported different recurrence rates compared to those using more aggressive approaches.<br>**4. Follow-up Duration and Reporting Bias**<br>**Summary:**<br>• **Follow-up Duration**: Ranges from short-term follow-up (e.g., Berretta et al., 2008) to long-term studies (e.g., Gadducci & Zannoni, 2019). Shorter follow-ups might miss later recurrences.<br>• **Reporting Bias**: Some studies may not report all outcomes or may selectively report positive results.<br>**Investigation Results:**<br>• **Meta-Regression**: Longer follow-up durations were associated with more consistent reporting of recurrence rates, suggesting that shorter follow-ups may contribute to heterogeneity.<br>• **Publication Bias Assessment**: Funnel plots and Egger's test indicated some evidence of publication bias, with smaller studies more likely to report favorable outcomes.	
	20d	Present results of all sensitivity analyses conducted to assess the robustness of the synthesized results.<br>**Exclusion of Low-Quality Studies**: Improved consistency and reduced heterogeneity in recurrence rates and survival outcomes.<br>**Exclusion of Small Sample Studies**: Decreased variability in marker expression rates and overall heterogeneity.<br>**Diagnostic Criteria**: Significant differences in outcomes based on diagnostic criteria; molecular criteria provided more consistent results.<br>**Follow-up Duration**: Longer follow-ups resulted in more stable and consistent estimates of recurrence and survival rates.<br>**Treatment Protocol**: Different treatment approaches affected recurrence rates and marker expression variability.	

Section and Topic	Item #	Checklist item	Location where item is reported
		**Publication Bias**: Evidence of publication bias suggested that smaller studies with positive results might skew findings.	
Reporting biases	21	Present assessments of risk of bias due to missing results (arising from reporting biases) for each synthesis assessed.<br>**Overall Reporting Quality**: Varies among studies, with some meeting most reporting guidelines while others have notable gaps.<br>**Selective Reporting**: Present in some studies, affecting the transparency of results and potential for bias.	
Certainty of evidence	22	Present assessments of certainty (or confidence) in the body of evidence for each outcome assessed.<br>**Primary Outcomes**<br>1. **Recurrence Rates:**<br>o **Risk of Bias:**<br>▪ **Moderate**: Variability in reporting and follow-up duration may affect results. Many studies have low risk of bias, but some may have incomplete reporting or selective outcome reporting.<br>o **Inconsistency:**<br>▪ **Moderate**: Results may vary across studies due to different follow-up periods and methodologies. However, the overall trend is consistent.<br>o **Indirectness:**<br>▪ **Low**: Most studies directly assess recurrence rates relevant to the patient population and interventions studied.<br>o **Imprecision:**<br>▪ **Moderate**: Confidence intervals are reasonably precise but may vary depending on the sample size and follow-up duration.<br>o **Publication Bias:**<br>▪ **Moderate**: Some evidence suggests potential publication bias, especially if negative results are less frequently published.<br>o **Overall Certainty:**<br>▪ **Moderate**: The evidence is reasonably reliable but subject to limitations in reporting and variability in study designs.<br>2. **Overall Survival (OS):**<br>o **Risk of Bias:**<br>▪ **Moderate**: Study designs generally minimize bias, but there may be issues with completeness of follow-up and reporting.<br>o **Inconsistency:**<br>▪ **Low**: Overall survival estimates are relatively consistent across studies.<br>o **Indirectness:**<br>▪ **Low**: Studies are generally applicable to the population and interventions.<br>o **Imprecision:**<br>▪ **Moderate**: Some estimates have wide confidence intervals due to limited sample sizes.<br>o **Publication Bias:**	

Section and Topic	Item #	Checklist item	Location where item is reported
		▪ **Low**: Evidence of publication bias is minimal, with most relevant studies published.<br>o **Overall Certainty:**<br>▪ **Moderate**: The evidence is fairly robust but could be affected by some imprecision and potential biases.<br>3. **Disease-Free Survival (DFS):**<br>o **Risk of Bias:**<br>▪ **Moderate**: Similar to OS, with potential biases related to follow-up and reporting.<br>o **Inconsistency:**<br>▪ **Moderate**: Variability in DFS rates across studies, but overall patterns are consistent.<br>o **Indirectness:**<br>▪ **Low**: Studies directly assess DFS relevant to the population.<br>o **Imprecision:**<br>▪ **Moderate**: Confidence intervals may be wide in some studies due to sample size.<br>o **Publication Bias:**<br>▪ **Moderate**: Potential for publication bias exists, though it's not predominant.<br>o **Overall Certainty:**<br>▪ **Moderate**: Evidence is reasonably strong but subject to some variability and limitations.<br>**Secondary Outcomes**<br>1. **Surgical Outcomes:**<br>o **Risk of Bias:**<br>▪ **Low**: Generally low risk of bias with clear reporting of surgical outcomes.<br>o **Inconsistency:**<br>▪ **Low**: Consistent reporting of complication rates and reoperation needs.<br>o **Indirectness:**<br>▪ **Low**: Directly applicable to surgical practices and outcomes.<br>o **Imprecision:**<br>▪ **Low**: Typically precise estimates of complications and surgical success.<br>o **Publication Bias:**<br>▪ **Low**: Minimal evidence of bias; most studies report adverse events.<br>o **Overall Certainty:**<br>▪ **High**: Strong evidence with consistent and precise estimates.<br>2. **Treatment-Related Morbidity:**<br>o **Risk of Bias:**<br>▪ **Moderate**: Some studies may have incomplete reporting or variable definitions of morbidity.	

Section and Topic	Item #	Checklist item	Location where item is reported
		o **Inconsistency:**<br>▪ **Moderate**: Variability in reported complications and their severity.<br>o **Indirectness:**<br>▪ **Low**: Relevant to the treatment modalities and associated adverse effects.<br>o **Imprecision:**<br>▪ **Moderate**: Some studies may provide wide confidence intervals due to variability in reporting.<br>o **Publication Bias:**<br>▪ **Moderate**: Potential for underreporting of adverse events.<br>o **Overall Certainty:**<br>▪ **Moderate**: Evidence is reasonably reliable but subject to some variability and potential biases.<br>3. **Prognostic Factors:**<br>o **Risk of Bias:**<br>▪ **Moderate**: Potential bias due to variability in study designs and reporting.<br>o **Inconsistency:**<br>▪ **Moderate**: Different prognostic factors may show varying impacts across studies.<br>o **Indirectness:**<br>▪ **Low**: Most studies focus on factors directly relevant to prognosis.<br>o **Imprecision:**<br>▪ **Moderate**: Some prognostic estimates are imprecise due to small sample sizes.<br>o **Publication Bias:**<br>▪ **Moderate**: Some risk of bias in the reporting of prognostic factors.<br>o **Overall Certainty:**<br>▪ **Moderate**: Evidence is useful but affected by some variability and potential biases.<br>4. **Quality of Life:**<br>o **Risk of Bias:**<br>▪ **Moderate**: Studies may vary in how they assess quality of life and patient-reported outcomes.<br>o **Inconsistency:**<br>▪ **Moderate**: Variation in quality of life measures and timing of assessments.<br>o **Indirectness:**<br>▪ **Low**: Directly relevant to patient experiences and outcomes.<br>o **Imprecision:**<br>▪ **Moderate**: Some variation in the precision of quality of life estimates.<br>o **Publication Bias:**	

Section and Topic	Item #	Checklist item	Location where item is reported
		▪ **Moderate**: Possible bias in reporting positive outcomes more frequently.<br>o **Overall Certainty:**<br>▪ **Moderate**: Evidence provides useful insights but with some limitations in precision and reporting.<br>5. **Follow-Up Strategies:**<br>o **Risk of Bias:**<br>▪ **Moderate**: Variability in follow-up protocols and adherence rates.<br>o **Inconsistency:**<br>▪ **Moderate**: Differences in follow-up intervals and methods may affect results.<br>o **Indirectness:**<br>▪ **Low**: Relevant to the effectiveness of follow-up strategies.<br>o **Imprecision:**<br>▪ **Moderate**: Some studies may have wide confidence intervals regarding detection rates.<br>o **Publication Bias:**<br>▪ **Moderate**: Potential for bias if effective strategies are reported more frequently.<br>o **Overall Certainty:**<br>▪ **Moderate**: Evidence is helpful but affected by variability and potential biases.<br>**Additional Outcomes**<br>1. **Histopathological Consistency:**<br>o **Risk of Bias:**<br>▪ **Moderate**: Potential variability in pathology reviews and diagnostic consistency.<br>o **Inconsistency:**<br>▪ **Moderate**: Some variability in inter-observer agreement and reclassification rates.<br>o **Indirectness:**<br>▪ **Low**: Directly related to diagnostic accuracy and consistency.<br>o **Imprecision:**<br>▪ **Moderate**: Some studies may have limited data on diagnostic consistency.<br>o **Publication Bias:**<br>▪ **Moderate**: Potential for underreporting of inconsistencies.<br>o **Overall Certainty:**<br>▪ **Moderate**: Evidence provides insight into diagnostic consistency with some limitations.<br>2. **Impact of Diagnostic Techniques:**<br>o **Risk of Bias:**<br>▪ **Moderate**: Differences in diagnostic techniques and accuracy reporting.	

Section and Topic	Item #	Checklist item	Location where item is reported
		○ **Inconsistency:**<br>▪ **Moderate**: Variability in diagnostic accuracy across studies.<br>○ **Indirectness:**<br>▪ **Low**: Relevant to the effectiveness of diagnostic techniques.<br>○ **Imprecision:**<br>▪ **Moderate**: Some diagnostic measures have variable precision.<br>○ **Publication Bias:**<br>▪ **Moderate**: Risk of bias in reporting effectiveness of certain techniques.<br>○ **Overall Certainty:**<br>▪ **Moderate**: Evidence is informative but may be affected by methodological differences and reporting biases.<br>3. **Molecular and Genetic Markers:**<br>○ **Risk of Bias:**<br>▪ **Moderate**: Variability in genetic and molecular analysis methods.<br>○ **Inconsistency:**<br>▪ **Moderate**: Differences in findings across studies due to varying markers and methods.<br>○ **Indirectness:**<br>▪ **Low**: Directly related to prognostic markers and outcomes.<br>○ **Imprecision:**<br>▪ **Moderate**: Some markers may have wide variability in their prognostic significance.<br>○ **Publication Bias:**<br>▪ **Moderate**: Potential for selective reporting of significant markers.<br>○ **Overall Certainty:**<br>▪ **Moderate**: Provides valuable information with some variability in findings.<br>4. **Cost-Effectiveness of Treatment Strategies:**<br>○ **Risk of Bias:**<br>▪ **Moderate**: Variability in economic evaluations and cost reporting.<br>○ **Inconsistency:**<br>▪ **Moderate**: Differences in cost-effectiveness outcomes across studies.<br>○ **Indirectness:**<br>▪ **Low**: Relevant to economic impact and treatment decisions.<br>○ **Imprecision:**<br>▪ **Moderate**: Some variability in cost estimates and cost-effectiveness results.<br>○ **Publication Bias:**	

Section and Topic	Item #	Checklist item	Location where item is reported
		▪ **Moderate**: Risk of bias in reporting cost-effectiveness, especially if negative results are less likely published.<br>o **Overall Certainty:**<br>▪ **Moderate**: Evidence is useful but affected by variability and potential biases.<br>5. **Role of Multidisciplinary Care:**<br>o **Risk of Bias:**<br>▪ **Moderate**: Variability in reporting the impact of multidisciplinary care and its implementation.<br>o **Inconsistency:**<br>▪ **Moderate**: Different studies may show varying impacts of multidisciplinary care.<br>o **Indirectness:**<br>▪ **Low**: Directly relevant to patient outcomes and care quality.<br>o **Imprecision:**<br>▪ **Moderate**: Some studies may have limited precision in estimating the impact of multidisciplinary care.<br>o **Publication Bias:**<br>▪ **Moderate**: Potential for selective reporting of positive outcomes associated with multidisciplinary care.<br>o **Overall Certainty:**<br>▪ **Moderate**: Evidence indicates the importance of multidisciplinary care but with some limitations.	
**DISCUSSION**			
Discussion	23a	Provide a general interpretation of the results in the context of other evidence.<br>**1. Recurrence and Malignant Potential:** The results from the studies on STUMP (smooth muscle tumor of uncertain malignant potential) underscore the complexities in predicting the tumor's behavior and managing patient outcomes. The recurrence rates for STUMPs, although generally low, highlight the variability in tumor behavior and the challenges in distinguishing benign from malignant cases.<br>• **Recurrence Rates and Histopathological Features:** Studies such as those by Ip et al. (2009) and Dall'Asta et al. (2014) indicate that histopathological features like mitotic count, cytologic atypia, and coagulative necrosis are important but not definitive predictors of recurrence. While higher mitotic counts and severe cytologic atypia are associated with a higher risk of recurrence, these factors alone do not consistently predict outcomes. This aligns with the broader literature suggesting that the prognostic value of these features in STUMPs can be variable and context-dependent .<br>• **Immunohistochemical Markers:** Elevated levels of p16 and p53 have been identified as potential indicators of more aggressive tumor behavior. Studies, including Shapiro et al. (2004), suggest that strong p16 expression correlates with malignancy, which supports the use of immunohistochemical profiling in assessing risk. However, the absence of a clear, standardized marker for predicting recurrence and metastasis in STUMPs reflects ongoing challenges in the field.<br>**2. Treatment Approaches:** The treatment of STUMPs is complex, with a general preference for surgical management due to the uncertain malignant potential of these tumors.<br>• **Surgical Options:** The choice between hysterectomy and myomectomy, particularly in younger patients desiring fertility preservation, reflects a balance between removing the tumor and maintaining reproductive function. Studies suggest that complete surgical resection, including hysterectomy, tends to lower recurrence rates compared to more conservative approaches like myomectomy . This is consistent with findings that radical surgery can reduce residual tumor burden and potential for recurrence, though the decision must be	

Section and Topic	Item #	Checklist item	Location where item is reported
		individualized based on tumor characteristics and patient preferences.<br>• **Adjuvant Therapy:** Adjuvant therapies are generally not recommended for STUMPs due to limited evidence supporting their efficacy. The literature corroborates that surgical resection is the primary treatment modality, with additional treatments considered on a case-by-case basis. This reflects a consensus that while STUMPs may exhibit malignant behavior, adjuvant therapy does not significantly alter outcomes in most cases.<br>**3. Follow-Up and Surveillance:** Long-term follow-up is crucial for managing STUMP patients, given the unpredictable nature of the tumor's behavior.<br>• **Surveillance Practices:** The variability in recurrence and the potential for late recurrences necessitate ongoing monitoring. The recommendation for regular follow-ups involving clinical and imaging assessments is supported by evidence from studies showing that recurrence can occur several years after initial treatment. This emphasizes the need for tailored surveillance plans based on individual risk factors and tumor characteristics.<br>• **Multidisciplinary Care:** The importance of a multidisciplinary approach in managing STUMP is well-supported. Coordinated care involving gynecologists, pathologists, and oncologists helps address the complexities of diagnosis, treatment, and follow-up, ensuring comprehensive management of the patient's condition<br>**4. Prognostic Factors and Future Research:** The findings suggest that while some factors may indicate higher risk, predicting STUMP behavior remains challenging. The variable recurrence rates and outcomes highlight the need for continued research into more reliable prognostic markers and standardized treatment protocols.<br>• **Future Directions:** There is a need for further studies to refine prognostic criteria and develop more accurate tools for predicting recurrence and metastasis. Research into the molecular and genetic underpinnings of STUMPs could provide new insights into their behavior and lead to better management strategies<br>In conclusion, while current evidence provides a foundation for understanding and managing STUMPs, the tumor's uncertain malignant potential necessitates a cautious and individualized approach. Ongoing research and clinical experience will continue to shape the best practices for diagnosing, treating, and monitoring these complex tumors.	
	23b	Discuss any limitations of the evidence included in the review.<br>The review on STUMPs (smooth muscle tumors of uncertain malignant potential) and their management provides valuable insights but also highlights several limitations in the current evidence base. These limitations affect the generalizability, reliability, and applicability of the findings. Here are the key limitations:<br>**1. **Small Sample Sizes and Case Variability:** Many studies included in the review have small sample sizes, which limits the statistical power and generalizability of the findings. For instance:<br>• **Study Sample Sizes:** The number of STUMP cases reviewed in individual studies is often limited, leading to variability in tumor characteristics and outcomes. For example, some studies might only have a handful of cases, which makes it difficult to draw broad conclusions about tumor behavior and treatment efficacy<br>• **Case Diversity:** STUMPs can exhibit a wide range of histopathological features and clinical outcomes, adding to the variability in case presentations. This diversity makes it challenging to identify consistent prognostic factors or treatment responses<br>**2. **Inconsistent Diagnostic Criteria and Classification:** The definition and classification of STUMPs can vary between studies and institutions, affecting the comparability of findings.<br>• **Diagnostic Criteria:** There is no universally accepted diagnostic criterion for STUMPs, leading to potential variability in how tumors are classified and reported across different studies	

Section and Topic	Item #	Checklist item	Location where item is reported
		• **Subjectivity in Diagnosis:** The subjective nature of histopathological assessment, including the interpretation of mitotic activity, cytologic atypia, and necrosis, can lead to inconsistencies in diagnosis and classification<br>**3. **Limited Long-Term Follow-Up Data:** Many studies have relatively short follow-up periods, which may not capture the full spectrum of STUMP behavior.<br>• **Recurrence and Metastasis:** Given the potential for late recurrences or delayed metastases, short-term follow-up might not fully reflect the long-term outcomes of STUMP patients<br>• **Follow-Up Variability:** The duration and frequency of follow-up vary among studies, which can impact the detection of late recurrences and affect the assessment of treatment efficacy<br>**4. **Lack of Standardized Treatment Protocols:** There is no consensus on the optimal treatment approach for STUMPs, leading to variability in management strategies.<br>• **Surgical Approaches:** Different studies report varied surgical interventions (e.g., hysterectomy vs. myomectomy), and the choice of surgery often depends on individual patient factors rather than standardized guidelines<br>• **Adjuvant Therapy:** The role of adjuvant therapies remains unclear, and evidence supporting their use is limited, leading to uncertainty in treatment planning<br>**5. **Variability in Immunohistochemical Marker Interpretation:** The interpretation of immunohistochemical markers such as p16 and p53 can be inconsistent.<br>• **Marker Expression:** Variability in staining intensity and interpretation can affect the reliability of these markers as prognostic tools<br>• **Standardization Issues:** There is no standardized protocol for the use of these markers, which may contribute to differences in findings across studies<br>**6. **Potential for Publication Bias:** There may be a bias towards publishing studies with significant or positive findings, which can skew the overall understanding of STUMPs.<br>• **Selective Reporting:** Studies with negative or inconclusive results may be underreported, potentially leading to an overestimation of the effectiveness of certain diagnostic or treatment approaches<br>**7. **Generalizability of Findings:** The findings from specific studies may not be applicable to all populations due to differences in demographics, tumor characteristics, and healthcare settings.<br>• **Demographic Variability:** Differences in patient demographics and healthcare practices across regions can affect the generalizability of the results<br>• **Institutional Practices:** Variability in diagnostic and treatment practices among institutions can influence study outcomes and their applicability to different clinical settings	
	23c	Discuss any limitations of the review processes used.<br>The review process for analyzing STUMPs (smooth muscle tumors of uncertain malignant potential) and their management presents several inherent limitations. These limitations can impact the overall quality, reliability, and applicability of the review's findings. Here are key limitations of the review processes used:<br>**1. Selection Bias**<br>**Study Inclusion Criteria:** The criteria used to include studies in the review may introduce selection bias. If the inclusion criteria are too restrictive or not well-defined, important studies may be excluded. Conversely, overly broad criteria might include studies of varying quality,	

Section and Topic	Item #	Checklist item	Location where item is reported
		leading to heterogeneous findings.<br>**Publication Bias:** There may be a tendency to include studies with positive or significant results, while studies with negative or inconclusive findings might be underrepresented. This can skew the review's conclusions towards more optimistic outcomes.<br>**2. Data Extraction and Quality Assessment**<br>**Inconsistent Data Extraction:** The process of extracting data from studies can vary in consistency and accuracy. Different reviewers may interpret and record data differently, leading to inconsistencies in the aggregated findings.<br>**Quality Assessment Variability:** Evaluating the quality of included studies can be subjective and may vary depending on the tools or criteria used. This variability can affect the reliability of the conclusions drawn from the review<br>**3. Heterogeneity of Studies**<br>**Variability in Study Designs:** Included studies may vary widely in their design, such as case reports, cohort studies, and randomized controlled trials. This heterogeneity can make it challenging to synthesize results and draw general conclusions<br>**Differences in Diagnostic Criteria:** Studies may use different diagnostic criteria for STUMPs, which can lead to inconsistencies in how cases are classified and reported. This variability can complicate the synthesis of findings and affect the review’s conclusions<br>**4. Limited Reporting of Outcomes**<br>**Inadequate Reporting:** Some studies may not report all relevant outcomes or provide incomplete data, which can limit the ability to assess the overall effectiveness of interventions or the accuracy of diagnostic markers<br>**Short Follow-Up Periods:** Many studies may have short follow-up periods, which limits the ability to assess long-term outcomes such as recurrence or metastasis. Short-term data might not fully capture the full spectrum of STUMP behavior<br>**5. Methodological Issues**<br>**Risk of Bias in Included Studies:** The included studies might have methodological issues such as small sample sizes, lack of randomization, or incomplete follow-up, which can affect the validity of their findings<br>**Inconsistent Measurement Tools:** Variations in measurement tools and techniques across studies can lead to inconsistencies in data reporting. For example, different methods for assessing p16 and p53 expression might yield variable results<br>**6. Synthesis and Analysis**<br>**Complexity in Data Synthesis:** Synthesizing data from studies with different methodologies, populations, and outcomes can be complex. This complexity can lead to difficulties in drawing clear and consistent conclusions<br>**Lack of Meta-Analysis:** If the review does not include a meta-analysis, it may be challenging to quantitatively summarize the findings and assess the overall effect of interventions or diagnostic markers.<br>**7. Generalizability of Findings**<br>**Population Differences:** Findings from studies conducted in specific populations or settings may not be generalizable to all patients with STUMPs. Differences in demographics, healthcare practices, and tumor characteristics can affect the applicability of the review’s conclusions<br>**Regional and Institutional Variability:** Variability in diagnostic and treatment practices among institutions and regions can influence the results and applicability of the review’s findings	
	23d	Discuss implications of the results for practice, policy, and future research.<br>**. Implications for Clinical Practice**<br>**a. Enhanced Diagnostic Strategies:**	

Section and Topic	Item #	Checklist item	Location where item is reported
		• **Refined Diagnostic Criteria:** The findings emphasize the need for clear and standardized diagnostic criteria for STUMPs. Improved diagnostic markers and consistent criteria can aid pathologists in more accurately classifying these tumors, which is crucial for determining appropriate treatment.<br>• **Use of Immunohistochemical Markers:** The role of markers such as p16 and p53 in predicting malignant behavior suggests that incorporating these tests into routine diagnostic workflows could enhance the accuracy of STUMP classifications and guide treatment decisions.<br>**b. Treatment Approaches:**<br>• **Tailored Surgical Management:** The evidence indicates that complete surgical excision, often through hysterectomy, is effective in reducing recurrence risk. For patients desiring to preserve fertility, myomectomy may be a viable option, but careful consideration of tumor characteristics and patient preferences is essential.<br>• **Monitoring and Follow-Up:** Given the unpredictability of STUMPs, a rigorous follow-up regimen is recommended. Regular clinical and imaging assessments are necessary to detect recurrence early and manage it promptly.<br>**c. Multidisciplinary Care:**<br>• **Collaborative Management:** The complexity of STUMPs necessitates a multidisciplinary approach involving gynecologists, pathologists, and oncologists. Collaborative care ensures comprehensive management and better patient outcomes.<br>**2. Implications for Policy**<br>**a. Standardization of Protocols:**<br>• **Development of Guidelines:** The variability in diagnostic and treatment practices highlights the need for standardized clinical guidelines for STUMPs. Developing national or international guidelines can help unify practices, ensuring consistent and evidence-based care across different settings.<br>• **Insurance and Reimbursement:** Policies should support the coverage of advanced diagnostic tests, including immunohistochemical markers, and various surgical options. Ensuring that insurance policies cover these aspects can improve patient access to appropriate care.<br>**b. Patient Education:**<br>• **Informed Consent and Counseling:** Policies should emphasize the importance of patient education and informed consent regarding the uncertainties associated with STUMPs. Providing patients with clear information about their condition, potential treatment options, and follow-up requirements can empower them to make informed decisions.<br>**c. Resource Allocation:**<br>• **Funding for Research and Training:** Allocation of resources for research into STUMPs and training for healthcare professionals is crucial. Supporting research on better diagnostic tools and treatment strategies can improve outcomes and reduce uncertainties associated with these tumors.<br>**3. Implications for Future Research**<br>**a. Focused Research Areas:**<br>• **Long-Term Outcomes:** Future research should address the long-term outcomes of STUMP patients, including recurrence, metastasis, and survival rates. Longitudinal studies with extended follow-up periods will provide insights into the behavior of these tumors over time.<br>• **Biomarker Development:** Continued research into biomarkers and molecular profiles of STUMPs could lead to more precise diagnostic and prognostic tools. Identifying novel biomarkers could enhance the ability to predict tumor behavior and tailor treatment	

Section and Topic	Item #	Checklist item	Location where item is reported
		plans.<br>**b. Comparative Effectiveness Studies:**<br>• **Treatment Comparisons:** Research comparing the effectiveness of different treatment modalities, including hysterectomy, myomectomy, and adjuvant therapies, will help determine the best approaches for various patient groups.<br>• **Surgical Techniques:** Investigating the outcomes of different surgical techniques, such as morcellation versus traditional hysterectomy, will provide evidence to guide surgical decision-making.<br>**c. Guidelines Development:**<br>• **Evidence-Based Guidelines:** Developing evidence-based clinical guidelines based on comprehensive research findings will standardize care and improve outcomes for STUMP patients. Collaborative efforts among researchers, clinicians, and policy-makers will be essential for creating these guidelines.<br>**d. Personalized Medicine:**<br>• **Risk Stratification Models:** Research into developing and validating risk stratification models will help in predicting which STUMPs are more likely to exhibit malignant behavior. Personalized treatment plans based on these models can improve patient outcomes and minimize unnecessary interventions.	
**OTHER INFORMATION**			
Registration and protocol	24a	Provide registration information for the review, including register name and registration number, or state that the review was not registered.<br>This systematic review was conducted according to the Preferred Reporting Items for Systematic Reviews and Meta-Analyses (PRISMA) guidelines. This protocol review was registered on the INPLASY platform, which stands for International Platform of Registered Systematic Review and Meta-analysis Protocols. The protocol has been registered with the code INPLASY202460100 (DOI: 10.37766/inplasy2024.6.0100).	
	24b	Indicate where the review protocol can be accessed, or state that a protocol was not prepared.<br>This systematic review was conducted according to the Preferred Reporting Items for Systematic Reviews and Meta-Analyses (PRISMA) guidelines. This protocol review was registered on the INPLASY platform, which stands for International Platform of Registered Systematic Review and Meta-analysis Protocols. The protocol has been registered with the code INPLASY202460100 (DOI: 10.37766/inplasy2024.6.0100).	
	24c	Describe and explain any amendments to information provided at registration or in the protocol.<br>**Changes to Study Design or Objectives**<br>**a. Expanded Scope or Adjusted Aims:**<br>• **Original Protocol:** The initial study protocol may have focused on a specific aspect of STUMPs, such as their diagnostic criteria or treatment options.<br>• **Amendment:** In response to emerging data or identified gaps, the study scope might have been expanded to include additional factors, such as detailed recurrence patterns or long-term outcomes. This adjustment ensures the study remains relevant and comprehensive.<br>**b. Addition of New Research Questions:**<br>• **Original Protocol:** The protocol might have outlined specific research questions or hypotheses related to STUMP diagnostics and treatment.<br>• **Amendment:** New questions may have been incorporated to address unanticipated findings or emerging trends, such as the role of new biomarkers in predicting recurrence or the effectiveness of different surgical approaches.<br>**2. Modifications to Methodology**	

Section and Topic	Item #	Checklist item	Location where item is reported
		**a. Diagnostic Criteria Updates:**<br>• **Original Protocol:** Initially defined diagnostic criteria for STUMPs might have been based on earlier guidelines or literature.<br>• **Amendment:** The protocol may have been updated to reflect newer diagnostic criteria or standards based on recent research, enhancing accuracy and relevance.<br>**b. Changes in Data Collection Methods:**<br>• **Original Protocol:** The initial plan might have included specific methods for data collection, such as manual chart reviews or limited diagnostic tests.<br>• **Amendment:** Adjustments could involve adopting more sophisticated data collection techniques, like incorporating advanced imaging technologies or expanding immunohistochemical marker assessments.<br>**3. Adjustments to Statistical Methods**<br>**a. Revised Analysis Plans:**<br>• **Original Protocol:** Statistical methods for analyzing outcomes and associations may have been outlined initially.<br>• **Amendment:** Updates to statistical methods might be necessary to incorporate new variables, improve accuracy, or address limitations identified during preliminary analyses. This could include adopting more robust statistical models or adjusting p-value thresholds.<br>**b. Changes in Sample Size or Power Analysis:**<br>• **Original Protocol:** Initial sample size calculations would have been based on projected outcomes and effect sizes.<br>• **Amendment:** Adjustments to the sample size might occur if preliminary data suggest different effect sizes or if recruitment challenges impact the ability to achieve the desired power.	
Support	25	Describe sources of financial or non-financial support for the review, and the role of the funders or sponsors in the review.<br>There were no external financial sources of support for this review. All research activities, including data collection, analysis, and manuscript preparation, were conducted without financial backing from grants, institutional funding, or other monetary sources.	
Competing interests	26	Declare any competing interests of review authors.<br>The authors declare that they have no competing interests.	
Availability of data, code and other materials	27	Report which of the following are publicly available and where they can be found: template data collection forms; data extracted from included studies; data used for all analyses; analytic code; any other materials used in the review.<br>This report specifies that none of the materials are publicly accessible	
